# Non-Hermitian systems based on 3D chirality enabled asymmetrical polarization switching and omni-polarizer action at an EP

**DOI:** 10.1038/s41377-025-01960-5

**Published:** 2025-11-18

**Authors:** Xianhui Fu, Hao Hu, Jiawei Zhang, Jiwei Qi, Sihao Zhang, Qiang Wu, Yao Lu, Zongqiang Chen, Jing Chen, Xuanyi Yu, Qian Sun, Jingjun Xu

**Affiliations:** Key Laboratory of Weak-Light Nonlinear Photonics, Ministry of Education, Tianjin Key Laboratory of Photonics and Technology of Information Science, TEDA Institute of Applied Physics and School of Physics, Nankai University, 300457 Tianjin, China

**Keywords:** Optical physics, Applied optics

## Abstract

Asymmetric mode/state switching and omni-polarizer action have demonstrated application potential and can be realized in non-Hermitian systems, which required a slow encircling process in the non-Hermitian parameter space in general. Is it possible to achieve the above functions only at an exceptional point (EP) without the encircling process? Here, we propose constructing a non-Hermitian system using three-dimensional (3D) chiral materials to realize the above functions at an EP instead of through the encircling process. Our results show that the 3D chiral non-Hermitian system exhibits properties that are quite different from those of traditional non-Hermitian optical systems. In our system, the eigenstates are different when propagating forward and backward, thus enabling asymmetric state switching. At the EP, the degenerate eigenstates of forward and backward propagations of the system become mutually orthogonal, which enables the system to act as an omni-polarizer. Crucially, to validate our claims, we propose a straightforward and widely applicable method to adjust a 3D chiral non-Hermitian system from a state far from an EP to near an EP. Based on this, we construct a free-space optical 3D chiral non-Hermitian system experimentally to directly observe the evolution of light polarization states near the EP. The experimental results prove that the proposed optical systems can achieve asymmetric state switching and omni polarizers at the EP, which is consistent with our theoretical expectations. Our work holds promise for various 3D chiral non-Hermitian optical applications, such as highly sensitive chirality measurements and polarization manipulation.

## Abstract (web summary)

A method is developed for adjusting non-Hermitian systems to a state near an exceptional point, enabling the experimental realization of an omni-polarizer in three-dimensional chiral non-Hermitian systems.

## Graphical abstract

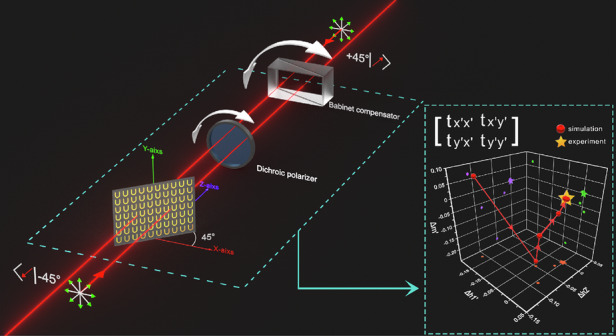

## Introduction

Recently, non-Hermitian systems have emerged as a fascinating area of research in physics, particularly non-Hermitian systems that can achieve asymmetric mode/state switching and omni-polarizer action, which have attracted much attention owing to their great potential in optical devices. This kind of polarizer, based on asymmetric mode/state switching, is fundamentally different from the traditional polarizer. It is that any input polarization state is output in a fixed polarization state in one direction and in an orthogonal state in the opposite direction. Initially, asymmetric mode/state switching^1–5^ and an omni-polarizer^6–9^ are generally implemented by dynamically encircling an EP. Subsequently, the latest theoretical and experimental evidence shows that asymmetric mode/state switching and omni-polarizer action can also be achieved without encircling an EP^10–15^; however, a slow encircling process in non-Hermitian parameter spaces is needed. This makes non-Hermitian systems more complex, and the proven transmission efficiency is very low because of path-related losses^1,3,4^. To overcome this limitation, chiral transmission by an open evolution trajectory has recently been proposed^16^. In addition, due to non-Hermitian induced nonadiabatic transitions (NATs)^17,18^, this dynamic encircling process can achieve the above function because it exhibits interesting chirality^6,19–24^. In this case, the encirclement proceeding in a clockwise (CW) manner or in a counterclockwise (ACW) manner will end in different final states. Since the dynamic encircling process makes the system more complex and dissipative, is it possible that we realize asymmetric mode/state switching and omni-polarizer action without the dynamic encircling process? Some innovative solutions are being developed to overcome the high loss problem of dynamic encircling associated with EP^16^.

It is well-known that EP is very unique and fascinating in non-Hermitian systems. Non-Hermitian systems have attracted extensive attention owing to their eigenvectors and their corresponding eigenvalues can be merged at a branch-point singularity, that is, an exceptional point (EP) in the parameter space^25–29^. EPs play a significant role in parity-time (PT) symmetric non-Hermitian optical systems, giving rise to various intriguing effects and phenomena. Some notable effects include loss-induced transparency^30^, unidirectional invisibility^31–33^, band merging^34^, single-mode lasing^35,36^, asymmetric diffraction^37^, nonreciprocal light propagation^38,39^, asymmetric modal conversion^40–42^, and novel phase transition^43^. These phenomena were realized at or close to an EP. Moreover, when a non-Hermitian system is at the EP, there is only one degenerate eigenstate, so any incoming state will evolve toward this degenerate eigenstate^44^. Consequently, taking advantage of the unique properties of the EP, can we achieve asymmetric mode/state switching and omni-polarizer action of non-Hermitian systems at an EP? In fact, to achieve asymmetric mode/state switching and omni-polarizers at the EP, the degenerate eigenstates of non-Hermitian systems need to be perpendicular to each other in forward and backward propagations. This requires that the parameters of non-Hermitian systems differ between forward and backward propagations. To the best of our knowledge, optical systems based on 3D chirality exhibit different Jones matrices when propagating forward and backward due to the principle of reciprocity^45^. Thus, it is possible to use 3D chiral materials to achieve the above functions at an EP without the encircling process^46^. In addition, compared with the implementation of the above functions in the dynamic encircling process, the implementation at the EP will have the advantage of low loss due to no parameter changes. However, there have been very few theoretical or experimental studies on this topic.

In this work, we introduce 3D chirality into a non-Hermitian system and explore its unique properties in detail. Initially, we derive the eigenvalues and eigenstates of the system for forward and backward propagations. Subsequently, we discuss the evolution of the eigenstates of the system during forward and backward propagations with changes in the system parameters. We further analyze the forward and backward Jones matrices of the system. Then, the evolution of light polarization states during forward and backward propagations is discussed for several systems with specific parameters. The theoretical analysis results show that the eigenstates for forward and backward propagations are different because of the different permittivity tensors, which leads to the asymmetric polarization evolution of light in 3D chiral non-Hermitian systems. Additionally, at the EP, the system has orthogonal degenerate eigenstates when propagating forward and backward, which can be regarded as omni-polarizer action. To further investigate the system, we propose a tunable and widely applicable experimental method that can adjust a 3D chiral non-Hermitian system from states far away from an EP to a state at or near an EP. Moreover, we construct a simple free-space experimental setup that includes an extrinsic chiral metasurface, a dichroic material, and a Babinet compensator as a 3D chiral non-Hermitian system. And then, we adjust this experimental composite system to a state near an EP. Consequently, the dynamic evolution of several representative input polarization states in the experimental composite system near the EP is directly observed during forward and backward propagations. The experimental results agree very well with the theoretical calculation results at the EP. The experimental results confirm the correctness of our theoretical results, namely, that asymmetric state switching and omni-polarizer action can be achieved in 3D chiral non-Hermitian systems.

## Results

It is well known that 3D chiral materials exhibit optical rotation and circular dichroism (CD). In general, chiral optical rotation and circular dichroism exist simultaneously. Here, we construct a general 3D chiral non-Hermitian system by introducing 3D chiral optical rotation and circular dichroism into a non-Hermitian system. In recent years, Fan et al. have proposed a very practical and clear method for describing the polarization state evolution of light in non-Hermitian media on the Poincaré sphere^43,44,47^. To show the properties of the general 3D chiral non-Hermitian system more simply and clearly, we adopt the analysis method of the literature^44^, that is, assume that the permeability is 1, and that the anisotropy is reflected only in the permittivity tensor matrix. In addition, to achieve non-Hermitian properties of the system, we introduce equal gains and losses in the directions of the *x*- and *y*-axis (special linear dichroism, LD). Then, we also consider the existence of birefringence, and its principal axes are *x* and *y*-axis. Thus, the forward and backward permittivity tensors of the general 3D chiral non-Hermitian system can be set to^45,47^

1 $${\varepsilon }^{{{{f}}}_{1}}=\left(\begin{array}{ccc}{\varepsilon }_{0}+{{i}}\gamma -\rho & -{{i}}\mu +\chi & 0\\ {{i}}\mu -\chi & {\varepsilon }_{0}-{{i}}\gamma +\rho & 0\\ 0 & 0 & {\varepsilon }_{0}\end{array}\right)$$

and

2 $${\varepsilon }^{{{{b}}}_{1}}=\left(\begin{array}{ccc}{\varepsilon }_{0}+{{i}}\gamma -\rho & {{i}}\mu -\chi & 0\\ -{{i}}\mu +\chi & {\varepsilon }_{0}-{{i}}\gamma +\rho & 0\\ 0 & 0 & {\varepsilon }_{0}\end{array}\right)$$

where ${\varepsilon }_{0}$ is a positive real number and $\gamma$ and $\mu$ represent the amount of gain or loss and coupling (chiral optical rotation), respectively. $\rho$ is the birefringent term, and $\chi$ is the CD term for the proposed system. For a plane wave propagating in the *z* direction (Forward), the permittivity tensor vector can be expressed as

3 $${\varepsilon }^{{{{f}}}_{1}}=\left(\begin{array}{cc}{\varepsilon }_{0}+{{i}}\gamma -\rho & -{{i}}\mu +\chi \\ {{i}}\mu -\chi & {\varepsilon }_{0}-{{i}}\gamma +\rho \end{array}\right)$$

Then, two eigenvalues can be solved:

4 $${\varepsilon }_{\,\,\,1,2}^{{{{f}}}_{1}}={\varepsilon }_{0}\pm {{\lambda}}_{pt}^{\prime}$$

where ${{\lambda}}_{{{pt}}}^{\prime} =\sqrt{{(\rho -{{i}}\gamma )}^{2}-{(\chi -{{i}}\mu )}^{2}}$ and the corresponding two eigenstates are as follows:

5 $$|{\psi }_{\quad\,\,\pm }^{{{R,{f}}}_{1}}\rangle =\left(\begin{array}{c}1\\ \displaystyle\frac{{{i}}\gamma -\rho \mp \sqrt{{(\rho -{{i}}\gamma )}^{2}-{(\chi -{{i}}\mu )}^{2}}}{{{i}}\mu -\chi }\end{array}\right)$$

When light propagates in the *−z* direction (backward), the reciprocity theorem is applied, and the form of the permittivity tensor is as follows:

6 $${\varepsilon }^{{{{b}}}_{1}}=\left(\begin{array}{cc}{\varepsilon }_{0}+{{i}}\gamma -\rho & {{i}}\mu -\chi \\ -{{i}}\mu +\chi & {\varepsilon }_{0}-{{i}}\gamma +\rho \end{array}\right)$$

The corresponding eigenvalues and eigenstates are as follows:

7 $${\varepsilon }_{\,\,\,1,2}^{{{{b}}}_{1}}={\varepsilon}_{0}\pm {{\lambda}}_{{{pt}}}^{\prime}$$

8 $$|{\psi }_{\quad\,\,\pm }^{{{R,{b}}}_{1}}\rangle =\left(\begin{array}{c}1\\ \displaystyle\frac{{{i}}\gamma -\rho \mp \sqrt{{(\rho -{{i}}\gamma )}^{2}-{(\chi -{{i}}\mu )}^{2}}}{\chi -{{i}}\mu }\end{array}\right)$$

Here, the superscript *f* denotes forward propagation, *R* denotes right eigenstates, and the superscript *b* denotes backward propagation. The eigenvalues for forward and backward propagations are the same, whereas the eigenstates are different. This characteristic distinguishes such systems from other non-Hermitian media. Then, when $\rho =\chi ,\gamma =\mu$, EP occurs in both forward propagation and backward propagation, where the eigenstates coalesce into ${(1,1)}^{{{T}}}$ and ${(1,-1)}^{{{T}}}$ in the forward and backward directions, respectively. In addition, when $\rho =-\chi ,\gamma =-\mu$, EP also occurs in both forward propagation and backward propagation, where the eigenstates coalesce into ${(1,-1)}^{{{T}}}$ and ${(1,1)}^{{{T}}}$ in the forward and backward directions, respectively. This situation, $\rho =-\chi ,\gamma =-\mu$, is not discussed later in this paper. Therefore, the system can realize an omni-polarizer function.

The 3D chiral non-Hermitian systems described above are the general cases, but there are two special cases. One contains only LD and chiral optical rotation without birefringence and CD. The forward and backward permittivity tensors in the system are shown below:

9 $${\varepsilon }^{{{{f}}}_{2}}=\left(\begin{array}{cc}{\varepsilon }_{0}+{{i}}\gamma & -{{i}}\mu \\ {{i}}\mu & {\varepsilon }_{0}-{{i}}\gamma \end{array}\right)$$

The corresponding eigenvalues and eigenstates are as follows:

10 $${\varepsilon }_{\,\,\,1,2}^{{{{f}}}_{2}}={\varepsilon}_{0}\pm {{\lambda}}_{{{pt}}}^{\prime\prime}$$

11 $$|{\psi }_{\quad\,\,\pm}^{{{R,{f}}}_{2}}\rangle =\left(\begin{array}{c}1\\ \displaystyle\frac{\gamma }{\mu }\pm {{i}}\sqrt{1-{\left(\frac{\gamma }{\mu }\right)}^{2}}\end{array}\right)$$

where $${{\lambda}}_{{{pt}}}^{\prime\prime} =\sqrt{{\mu }^{2}-{\gamma }^{2}}$$, and

12 $${\varepsilon }^{{{{b}}}_{2}}=\left(\begin{array}{cc}{\varepsilon }_{0}+{{i}}\gamma & {{i}}\mu \\ -{{i}}\mu & {\varepsilon }_{0}-{{i}}\gamma \end{array}\right)$$

The corresponding eigenvalues and eigenstates are as follows:

13 $${\varepsilon }_{\,\,\,1,2}^{{{{b}}}_{2}}={\varepsilon}_{0}\pm {\lambda}_{{{pt}}}^{\prime\prime}$$

14 $$|{\psi }_{\quad\,\,\pm }^{{{R,{b}}}_{2}}\rangle =\left(\begin{array}{c}1\\ -\displaystyle\frac{\gamma }{\mu }\mp {{i}}\sqrt{1-{\left(\frac{\gamma }{\mu }\right)}^{2}}\end{array}\right)$$

when $\gamma =\mu$, EP occurs in both forward propagation and backward propagation, the eigenstates coalesce into ${(1,1)}^{{{T}}}$ and ${(1,-1)}^{{{T}}}$ in the forward and backward directions, respectively.

The other special case contains only birefringence and CD without LD and chiral optical rotation. Similarly, the forward and backward permittivity tensors of the system can be given

15 $${\varepsilon }^{{{{f}}}_{3}}=\left(\begin{array}{cc}{\varepsilon }_{0}-\rho & \chi \\ -\chi & {\varepsilon }_{0}+\rho \end{array}\right)$$

The corresponding eigenvalues and eigenstates are as follows:

16 $${\varepsilon }_{\,\,\,1,2}^{{{{f}}}_{3}}={\varepsilon}_{0}\pm {\lambda}_{{{pt}}}^{\prime\prime\prime }$$

17 $$|{\psi }_{\quad\,\,\pm }^{{{R,{f}}}_{3}}\rangle =\left(\begin{array}{c}1\\ \displaystyle\frac{\rho }{\chi }\pm {{i}}\sqrt{1-{\left(\frac{\rho }{\chi }\right)}^{2}}\end{array}\right)$$

where $${\lambda}_{{{pt}}}^{\prime\prime\prime}=\sqrt{{\rho }^{2}-{\chi }^{2}}$$, and

18 $${\varepsilon }^{{{{b}}}_{3}}=\left(\begin{array}{cc}{\varepsilon }_{0}-\rho & -\chi \\ \chi & {\varepsilon }_{0}+\rho \end{array}\right)$$

The corresponding eigenvalues and eigenstates are as follows:

19 $${\varepsilon }_{\,\,\,\,1,2}^{{{{b}}}_{3}}={\varepsilon}_{0}\pm {\lambda}_{{{pt}}}^{\prime\prime\prime}$$

20 $$|{\psi }_{\quad\,\,\pm }^{{{R,{b}}}_{3}}\rangle =\left(\begin{array}{c}1\\ -\displaystyle\frac{\rho }{\chi }\mp {i}\sqrt{1-{\left(\frac{\rho }{\chi }\right)}^{2}}\end{array}\right)$$

when $\rho =\chi$, EP occurs in both forward propagation and backward propagation, the eigenstates also coalesce into ${(1,1)}^{{{T}}}$ and ${(1,-1)}^{{{T}}}$ in the forward and backward directions, respectively. Further, regarding the evolution of the eigenstates of the 3D chiral non-Hermitian system in two special cases with the change of parameters and the evolution of the polarization states under several specific parameters, specifically described in S1 of the supplementary material.

**Fig. 1:**
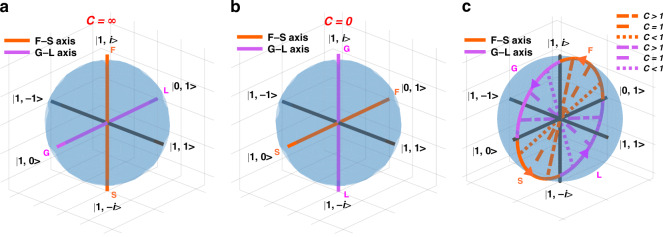
Discussion on the G-L axis and F-S axis of different 3D chiral non-Hermitian systems. **a** The Poincaré sphere shows the coordinates of the G–L axis (purple) and the F–S axis (orange) of forward propagation of a special 3D non-Hermitian system without birefringence and CD. **b** The Poincaré sphere shows the coordinates of the G–L axis (purple) and the F–S axis (orange) of forward propagation of a special 3D non-Hermitian system without LD and optical rotation. **c** The poincaré sphere shows how the G–L axis (purple) and F–S axis (orange) of a general 3D chiral non-Hermitian system change with different *C* values as it propagates forward

The general 3D chiral non-Hermitian system can be considered as a superposition of the two special cases mentioned above. In order to understand 3D chiral non-Hermitian systems better, here we discuss a series of cases where two special cases are superimposed in a certain proportion. Here, we define the ratios of the parameters in Eq. (1) as $\gamma /\chi =C=\mu /\rho$. When $C=\infty$ ($\chi =0,\rho =0$), the general system degenerates to the special case that only LD and optical rotation exist, as shown in Eq. (9). When $C=0$ ($\gamma =0,\mu =0$), the general system degenerates to the special case that only birefringence and CD exist, as shown in Eq. (15). The gain–loss (G–L) axis (purple) and fast–slow (F–S) axis (orange) of the two special cases above as shown on the Poincaré sphere in Fig. 1a and b. According to the definition of ratio *C*, the G–L axis and F–S axis evolve with the change of *C*, as shown in Fig. 1c. As the ratio *C* increases from zero, the G–L axis evolves from the (1, *i*)−(1, −*i*) to the (1, 0)−(0, 1) axis, and the F–S axis evolves from the (0, 1)−(1, 0) axis to the (1, *i*)−(1, −*i*) axis. When *C* tends to infinity, the G–L axis evolves into the (1, 0)−(0, 1) axis, and the F–S axis evolves into the (1, *i*)−(1, −*i*) axis. When propagating backward, the situation is similar to that of forward propagation.

**Fig. 2:**
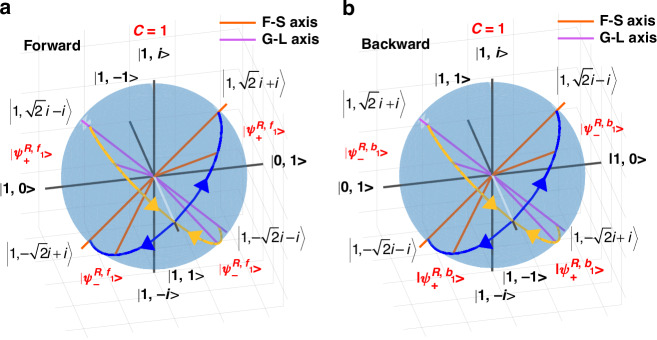
The evolution of eigenstates of a general 3D chiral non-Hermitian system. Poincaré spheres show the evolution of eigenstates of light passing through a general 3D chirality non-Hermitian system with $C=1$ ($\chi =\gamma ,\rho =\mu$) in both the forward (**a**) and backward (**b**) directions. Where the yellow and blue trajectories respectively represent the evolution of the eigenstates with different $\left|\frac{\gamma }{\mu }\right|$

Next, we take $C=1$ ($\chi =\gamma ,\rho =\mu$) as an example to describe the evolution of eigenstates of the general 3D chiral non-Hermitian systems with variation of parameters and the evolution of polarization states under several specific parameters. Figure 2a and b show the eigenstate evolution of the general 3D chiral non-Hermitian system during forward and backward propagation with the change of $\left|\frac{\gamma }{\mu }\right|$, when $C=1$ ($\chi =\gamma ,\rho =\mu$), respectively. The eigenstate evolution of the general 3D chiral non-Hermitian system is different in the forward and backward propagation processes. When $|\frac{\gamma }{\mu }|={\infty}\,(\gamma \ne 0,\mu =0)$, the eigenstates of forward propagation are ${(1,\sqrt{2}i-i)}^{{{T}}}$ and ${(1,-\sqrt{2}i-i)}^{{{T}}}$, where the gain eigenstate is ${(1,\sqrt{2}i-i)}^{{{T}}}$ and the loss eigenstate is ${(1,-\sqrt{2}i-i)}^{{{T}}}$^44^. However, for backward propagation, the eigenstates are ${(1,-\sqrt{2}i+i)}^{{{T}}}$ and ${(1,\sqrt{2}i+i)}^{T}$, where the gain eigenstate is ${(1,-\sqrt{2}i+i)}^{T}$ and the loss eigenstate is ${(1,i+\sqrt{2}i)}^{T}$. When $\left|\frac{\gamma }{\mu }\right| > 1$, with decreasing $\left|\frac{\gamma }{\mu }\right|$, the two eigenstates $|{\psi }_{\quad\,\,\pm }^{R,{f}_{1}}\rangle$ converge from ${(1,\sqrt{2}i-i)}^{T}$ and ${(1,-\sqrt{2}i-i)}^{T}$ to ${(1,1)}^{T}$, while $|{\psi }_{\quad\,\,\pm }^{R,{b}_{1}}\rangle$ converges from ${(1,-\sqrt{2}i+i)}^{T}$ and ${(1,\sqrt{2}i+i)}^{T}$ to ${(1,-1)}^{T}$, respectively, represented by the yellow trajectory on the sphere. When $\left|\frac{\gamma }{\mu }\right|=1$, that is, $\gamma =\mu =\rho =\chi$, the EP occurs in both the forward and backward propagation systems. The eigenstates of forward and backward propagation degenerate at ${(1,1)}^{T}$ and${(1,-1)}^{T}$, respectively. As $\left|\frac{\gamma }{\mu }\right|$ continues to decrease, when $\left|\frac{\gamma }{\mu }\right| < 1$, the eigenstates $|{\psi }_{\quad\,\,\pm }^{R,{f}_{1}}\rangle$ split from the ${(1,1)}^{T}$ to the ${(1,\sqrt{2}i+i)}^{T}$ and ${(1,-\sqrt{2}i+i)}^{T}$ states respectively, while $|{\psi }_{\quad\,\,\pm }^{R,{b}_{1}}\rangle$ split from the ${(1,-1)}^{T}$ to the ${(1,-\sqrt{2}i-i)}^{T}$ and ${(1,\sqrt{2}i-i)}^{T}$ states respectively, represented by the blue trajectory on the sphere. When $|\frac{\gamma }{\mu }|=0\,(\gamma =0,\mu \ne 0)$, the eigenstates of forward propagation are ${(1,\sqrt{2}i+i)}^{T}$ and ${(1,-\sqrt{2}i+i)}^{T}$, where the fast eigenstate is ${(1,\sqrt{2}i+i)}^{T}$ and the slow eigenstate is ${(1,-\sqrt{2}i+i)}^{T}$. However, for backward propagation, the eigenstate are ${(1,\sqrt{2}i-i)}^{T}$ and ${(1,-\sqrt{2}i-i)}^{T}$, where the fast eigenstate is ${(1,-\sqrt{2}i-i)}^{T}$ and the slow eigenstate is ${(1,\sqrt{2}i-i)}^{T}$. Hence, the eigenstate evolution process shows that the eigenstates of the proposed system are different for forward and backward propagation when chirality exists.

**Fig. 3:**
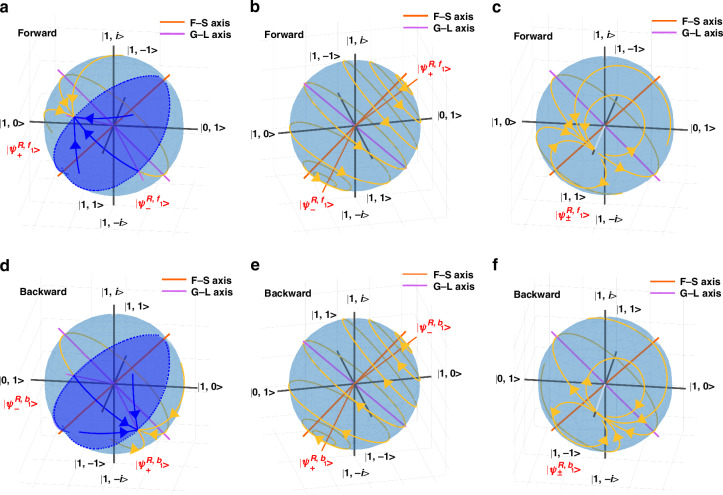
The light polarization state evolution passing through a general 3D chiral non-Hermitian system. Poincaré spheres describe the polarization dynamics of light passing through a general 3D chirality non-Hermitian system with $C=1$ ($\chi =\gamma ,\rho =\mu$) in both the forward and backward directions for a specific $\left|\frac{\gamma }{\mu }\right|$, where **a** and **d** correspond to $\left|\frac{\gamma }{\mu }\right|=\frac{2\sqrt{3}}{3}$, **b** and **e** correspond to $\left|\frac{\gamma }{\mu }\right|=\frac{1}{3}$, and **c** and **f** correspond to $\left|\frac{\gamma }{\mu }\right|=1$

Then, for several systems with a specific $\left|\frac{\gamma }{\mu }\right|$ when $C=1$ ($\chi =\gamma ,\rho =\mu$), the evolution of light polarization states during forward and backward propagation is analyzed. When forward propagation is considered, in the broken phase of $\left|\frac{\gamma }{\mu }\right|=\frac{2\sqrt{3}}{3}$, the eigenvalues are two different complex numbers in Eq. (4) and the corresponding eigenstates are two nonorthogonal elliptic polarizations that experience equal gain and loss. The polarization dynamics in this case are depicted on the Poincaré sphere, as shown in Fig. 3a. Since $|{\psi }_{\quad\,\,+}^{{{R,{f}}}_{1}}\rangle$ is the gain eigenstate, and $|{\psi }_{\quad\,\,-}^{{{R,{f}}}_{1}}\rangle$ is the loss eigenstate, any polarization eigenstate except the loss eigenstate $|{\psi }_{\quad\,\,-}^{{{R,{f}}}_{1}}\rangle$ will be amplified and rotated toward the gain eigenstate $|{\psi }_{\quad\,\,+}^{{{R,{f}}}_{1}}\rangle$, as described in the yellow and blue trajectories on the Poincaré sphere. Nevertheless, in the exact phase of $\left|\frac{\gamma }{\mu }\right|=\frac{1}{3}$, the eigenvalues are two different real numbers, and the corresponding eigenstates are two elliptically polarization states. The other polarization states precess in circles about nonorthogonal eigenstates $|{\psi }_{\quad\,\,\pm }^{{{R,{f}}}_{1}}\rangle$, as shown in Fig. 3b. For $\left|\frac{\gamma }{\mu }\right|=\frac{2\sqrt{3}}{3}$ in the backward propagation direction, the result is similar to that of forward propagation, with the exception of the loss eigenstate $|{\psi }_{\quad\,\,-}^{{{R,{b}}}_{1}}\rangle$, all polarization states are amplified toward the gain eigenstate $|{\psi }_{\quad\,\,+}^{{{R,{b}}}_{1}}\rangle$. However, a notable distinction arises from the fact that the forward and backward polarization eigenstates in our system are different, so the gain eigenstate $|{\psi }_{\quad\,\,+}^{{{R,{b}}}_{1}}\rangle$ and loss eigenstate $|{\psi }_{\quad\,\,-}^{{{R,{b}}}_{1}}\rangle$ are different from those of forward propagation, resulting in different evolution trajectory of the eigenstates compared with forward propagation, as shown in Fig. 3d. Moreover, for $\left|\frac{\gamma }{\mu }\right|=\frac{1}{3}$ in backward propagation, the result is also analogous to that in forward propagation. The polarization state precesses in circles around a pair of nonorthogonal states $|{\psi }_{\quad\,\,\pm }^{{{R,{b}}}_{1}}\rangle$. The yellow trajectory on the sphere in Fig. 3e is shown. Similarly, the direction of rotation of the polarization state is different from that of forward propagation. Equations (4) and (7) indicate that when $\left|\frac{\gamma }{\mu }\right|=1$, that is $\chi =\gamma =\rho =\mu$, EP occurs in both forward propagation and backward propagation, where the eigenstates coalesce into ${(1,1)}^{{{T}}}$ and ${(1,-1)}^{{{T}}}$ in the forward and backward directions, respectively. And no matter what polarization state is incident, the polarization of light will evolve toward the eigenstate, as shown in Fig. 3c and f. At this time, the polarization of light propagating forward and backward will evolve toward two vertically degenerate eigenstates. Therefore, we can conclude that due to the distinct eigenstates of forward and backward propagation of 3D chiral non-Hermitian systems, polarized light will experience different polarization evolutions when transmitted forward and backward, resulting in asymmetric state switching. When the 3D chiral non-Hermitian systems are at the EP, the light propagating forward and backward will end up in two orthogonal polarization states after a sufficiently long evolutionary distance. On this basis, an omni-polarizer is realized. These rules hold true for any *C* value. This provides a basis for our experimental implementation of a near EP in 3D chiral non-Hermitian systems.

**Fig. 4:**
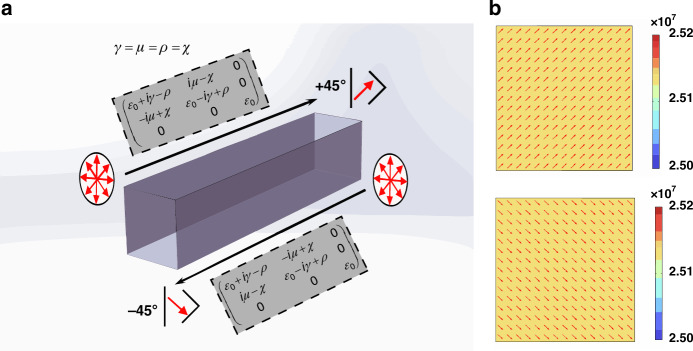
Simulation of the omni-polarizer achieved in general 3D chiral non-Hermitian system at an EP. **a** Schematic diagram of the omni-polarizer during forward and backward propagations. The gray area represents the permittivity tensors of a general 3D chiral non-Hermitian system with $C=1$ propagating forward and backward, where the direction-dependent polarization conversions are indicated by red arrows. **b** The corresponding two eigenpolarizations and their electric field polarization diagrams

Figure 4a shows that after propagation in a 3D chiral non-Hermitian system with a sufficiently long propagation distance, the output polarizations for an arbitrary input polarization state (300 input polarization states were simulated by COMSOL; the simulated settings are detailed in S2 of the supplementary material) evolve into the eigenstates ${(1,\pm 1)}^{{{T}}}$ for forward and backward propagation. All the electric field polarizations are almost the same, as illustrated in Fig. 4b (±45° linear polarization), which corresponds to the eigenstates ${(1,\pm 1)}^{{{T}}}$.

Furthermore, the Jones matrices corresponding to Eq. (1) are derived. From the relation between the incident wave ${E}_{{inc}}=[{E}_{x{\rm {I}}},{E}_{y{\rm {I}}}]$ and the transmitted wave ${E}_{{{tra}}}=[{E}_{x{\rm {T}}},{E}_{y{\rm {T}}}]$, the Jones matrices corresponding to ${\varepsilon }^{{{{f}}}_{1}}$ and ${\varepsilon }^{{{{b}}}_{1}}$ can be expressed using structural and material parameters as ${E}_{{{tra}}}^{{{{f}}}_{1}}={t}^{{{{f}}}_{1}}{E}_{{inc}}^{{{{f}}}_{1}}$ and ${E}_{{{tra}}}^{{{{b}}}_{1}}={t}^{{{{b}}}_{1}}{E}_{{{inc}}}^{{{{b}}}_{1}}$ respectively^47^,

21 $${t}^{{{{f}}}_{1}}=\left(\begin{array}{cc}{t}_{xx}^{{{{f}}}_{1}} & {t}_{xy}^{{{{f}}}_{1}}\\ {t}_{yx}^{{{{f}}}_{1}} & {t}_{yy}^{{{{f}}}_{1}}\end{array}\right)=\displaystyle\frac{1}{2}\left(\begin{array}{cc}({\varphi }_{1}+{\varphi }_{2})-\displaystyle\frac{\rho -{{i}}\gamma }{{\lambda_{pt} ^{\prime} }}({\varphi }_{1}-{\varphi }_{2}) & -i\displaystyle\frac{\mu +{{i}}\chi }{{\lambda_{pt} ^{\prime} }}({\varphi }_{1}-{\varphi }_{2})\\ {{i}}\displaystyle\frac{\mu +{\rm {i}}\chi }{{\lambda_{pt} ^{\prime} }}({\varphi }_{1}-{\varphi }_{2}) & ({\varphi }_{1}+{\varphi }_{2})+\displaystyle\frac{\rho -{{i}}\gamma }{{\lambda_{pt} ^{\prime} }}({\varphi }_{1}-{\varphi }_{2})\end{array}\right)$$

and

22 $${t}^{{{{b}}}_{1}}=\left(\begin{array}{cc}{t}_{xx}^{{{{b}}}_{1}} & {t}_{xy}^{{{{b}}}_{1}}\\ {t}_{yx}^{{{{b}}}_{1}} & {t}_{yy}^{{{{b}}}_{1}}\end{array}\right)=\displaystyle\frac{1}{2}\left(\begin{array}{cc}({\varphi }_{1}+{\varphi }_{2})-\displaystyle\frac{\rho -{{i}}\gamma }{{\lambda_{pt} ^{\prime} }}({\varphi }_{1}-{\varphi }_{2}) & {{i}}\displaystyle\frac{\mu +{{i}}\chi }{{\lambda_{pt} ^{\prime} }}({\varphi }_{1}-{\varphi }_{2})\\ -{{i}}\displaystyle\frac{\mu +{{i}}\chi }{{\lambda_{pt} ^{\prime} }}({\varphi }_{1}-{\varphi }_{2}) & ({\varphi }_{1}+{\varphi }_{2})+\displaystyle\frac{\rho -{{i}}\gamma }{{\lambda_{pt} ^{\prime} }}({\varphi }_{1}-{\varphi }_{2})\end{array}\right)$$

where ${\varphi }_{1}=\exp (-{\rm {i}}{\beta }_{1}d)$, ${\varphi }_{2}=\exp (-{\rm {i}}{\beta }_{2}d)$, ${\beta }_{1,2}=\frac{2\pi \sqrt{{\varepsilon }_{\,\,\,1,2}^{{{{f}}}_{1},{{{b}}}_{1}}}}{{\Lambda }_{0}}$, and ${\Lambda }_{0}$ is the wavelength selected under vacuum conditions in the experiment. When the general system is at an EP, $\rho =\chi ,\gamma =\mu$, the Jones matrices corresponding to ${\varepsilon }^{{{{f}}}_{1}}$ and ${\varepsilon }^{{{{b}}}_{1}}$ can be expressed as

23 $${t}^{{{{f}}}_{1},{{EP}}}=\left(\begin{array}{cc}{{i}}(\chi -{{i}}\mu )\displaystyle\frac{\pi d{{\varepsilon }_{0}}^{-\frac{1}{2}}}{{\Lambda }_{0}}{\varphi }_{0}+{\varphi }_{0} & -{{i}}(\chi -{{i}}\mu )\displaystyle\frac{\pi d{{\varepsilon }_{0}}^{-\frac{1}{2}}}{{\Lambda }_{0}}{\varphi }_{0}\\ {{i}}(\chi -{{i}}\mu )\displaystyle\frac{\pi d{{\varepsilon }_{0}}^{-\frac{1}{2}}}{{\Lambda }_{0}}{\varphi }_{0} & -{{i}}(\chi -{{i}}\mu )\displaystyle\frac{\pi d{{\varepsilon }_{0}}^{-\frac{1}{2}}}{{\Lambda }_{0}}{\varphi }_{0}+{\varphi }_{0}\end{array}\right)$$

and

24 $${t}^{{{{b}}}_{1},{{EP}}}=\left(\begin{array}{cc}{{i}}(\chi -{{i}}\mu )\displaystyle\frac{\pi d{{\varepsilon }_{0}}^{-\frac{1}{2}}}{{\Lambda }_{0}}{\varphi }_{0}+{\varphi }_{0} & {{i}}(\chi -{{i}}\mu )\displaystyle\frac{\pi d{{\varepsilon }_{0}}^{-\frac{1}{2}}}{{\Lambda }_{0}}{\varphi }_{0}\\ -{{i}}(\chi -{{i}}\displaystyle\mu )\frac{\pi d{{\varepsilon }_{0}}^{-\frac{1}{2}}}{{\Lambda }_{0}}{\varphi }_{0} & -{{i}}(\chi -{{i}}\mu )\displaystyle\frac{\pi d{{\varepsilon }_{0}}^{-\frac{1}{2}}}{{\Lambda }_{0}}{\varphi }_{0}+{\varphi }_{0}\end{array}\right)$$

respectively, where ${\varphi }_{0}=\exp (-{\rm {i}}{\beta }_{0}d)$ and ${\beta }_{0}=\frac{2\pi \sqrt{{\varepsilon }_{0}}}{{\varLambda }_{0}}$. To highlight the characteristics of the Jones matrix when the general system is at the EP, we rotate the coordinates 45° clockwise. In addition, to distinguish the coordinates before and after the rotation, we use $t=\{{t}_{ij}\}$ for the pre-rotation and $t^{\prime} =\{{t^{\prime} }_{i^{\prime} j^{\prime} }\}$ for the post-rotation. Then, Eqs. (21) and (22) can be rewritten as

25 $${t}^{{f^{\prime} }_{1}}=\left(\begin{array}{cc}{t}_{x^{\prime} x^{\prime} }^{{f^{\prime} }_{1}} & {t}_{x^{\prime} y^{\prime} }^{{f^{\prime} }_{1}}\\ {t}_{y^{\prime} x^{\prime} }^{{f^{\prime} }_{1}} & {t}_{y^{\prime} y^{\prime} }^{{f^{\prime} }_{1}}\end{array}\right)=\displaystyle\frac{1}{2}\left(\begin{array}{cc}{\varphi }_{1}+{\varphi }_{2} & \displaystyle\frac{(\rho -{{i}}\gamma )+(\chi -{{i}}\mu )}{\lambda_{pt}^{\prime}}({\varphi }_{1}-{\varphi }_{2})\\ \displaystyle\frac{(\rho -{{i}}\gamma )-(\chi -{{i}}\mu )}{{\lambda_{pt}^{\prime} }}({\varphi }_{1}-{\varphi }_{2}) & {\varphi }_{1}+{\varphi }_{2}\end{array}\right)$$

and

26 $${t}^{{b^{\prime} }_{1}}=\left(\begin{array}{cc}{t}_{x^{\prime} x^{\prime} }^{{b^{\prime} }_{1}} & {t}_{x^{\prime} y^{\prime} }^{{b^{\prime} }_{1}}\\ {t}_{y^{\prime} x^{\prime} }^{{b^{\prime} }_{1}} & {t}_{y^{\prime} y^{\prime} }^{{b^{\prime} }_{1}}\end{array}\right)=\displaystyle\frac{1}{2}\left(\begin{array}{cc}{\varphi }_{1}+{\varphi }_{2} & \displaystyle\frac{(\rho -{{i}}\gamma )-(\chi -{{i}}\mu )}{\lambda_{pt}^{\prime} }({\varphi }_{1}-{\varphi }_{2})\\ \displaystyle\frac{(\rho -{{i}}\gamma )+(\chi -{{i}}\mu )}{{\lambda_{pt}^{\prime} }}({\varphi }_{1}-{\varphi }_{2}) & {\varphi }_{1}+{\varphi }_{2}\end{array}\right)$$

Similarly, when the system is at an EP, the Jones matrices corresponding to ${\varepsilon }^{{f}_{1}}$ and ${\varepsilon }^{{b}_{1}}$ can be represented as

27 $${t}^{{f^{\prime} }_{1},{{EP}}}=\left(\begin{array}{cc}{\varphi }_{0} & -{{i}}(\chi -{{i}}\mu )\displaystyle\frac{2\pi d{{\varepsilon }_{0}}^{-\frac{1}{2}}}{{\Lambda }_{0}}{\varphi }_{0}\\ 0 & {\varphi }_{0}\end{array}\right)$$

28 $${t}^{{b^{\prime} }_{1},{{EP}}}=\left(\begin{array}{cc}{\varphi }_{0} & 0\\ -{{i}}(\chi -{{i}}\mu )\displaystyle\frac{2\pi d{{\varepsilon }_{0}}^{-\frac{1}{2}}}{{\Lambda }_{0}}{\varphi }_{0} & {\varphi }_{0}\end{array}\right)$$

The detailed derivation process is described in S3 of the supplementary material. The above results indicate that when 3D chiral non-Hermitian systems are at an EP, the forms of their forward and backward Jones matrices satisfy Eqs. (27) and (28), respectively. Thus, when the forward and backward Jones matrices satisfy Eqs. (27) and (28), the polarization evolution of the 3D chiral non-Hermitian systems can reach the EP^48^.

In addition, constructing a non-Hermitian system that achieves near an EP and direct observation of the evolution of the polarization states of light is desirable for the study of non-Hermitian systems^12^. By designing the metasurface, it is theoretically feasible to make a 3D chiral non-Hermitian system very close to the EP. However, the design and fabrication of this metasurface are very difficult and extremely costly. Therefore, a tunable and widely applicable experimental method is needed to implement adjusting non-Hermitian systems to a state at or near an EP. Here, we construct an experimental system in which we utilize a dichroic material and a Babinet compensator to tune a non-Hermitian system (including only an extrinsic chiral metasurface) to a state near the EP. The schematic diagram of the experimental setup is shown in Fig. 6a. In our experimental setup, 1550 nm wavelength light from a laser is passed through a polarizer and a broadband quarter-wave plate (Thorlabs) to form the required polarized light. Next, the polarized light is incident on a 3D chiral non-Hermitian system, which consists of a large-area extrinsic chiral metasurface (the structure parameters are shown in S4 of the supplementary materials), a dichroic material, and a Babinet compensator. Here, the large area of extrinsic chiral metasurface is employed to provide the main 3D chirality under oblique irradiation, as it can not only provide large chirality, but is also relatively easy to prepare^49^. In addition, the large-area extrinsic chiral metasurface also provides LD in the *x*- and *y*-directions. The dichroic material and Babinet compensator are employed to modulate the non-Hermitian system from states far away from an EP to near an EP (the selection of the dichroic material is detailed in S5 of the supplementary material). Finally, the transmitted light is detected by a polarimeter. In this experimental setup, we can adjust the angle ${\theta }_{{{D}}i}$ of the dichroic material (${\theta }_{{{D}}i}$ is the angle between the optical axis of a dichroic material and the *x*-polarization direction), the angle ${\theta }_{{{Bib}}}$ of the Babinet compensator (${\theta }_{{{Bib}}}$ is the angle between the optical axis of a Babinet compensator and the *x*-polarization direction), the loss coefficient ${\alpha }_{{{D}}i}$ of the dichroic material, and the birefringence coefficient ${\beta }_{{{Bib}}}$ of the Babinet compensator to ensure that the forward propagation and backward propagation Jones matrix of the entire composite system matches the form of Eqs. (27) and (28) (near an EP).

Next, we give a detailed description of the principle and process of adjusting the above experimental composite system. Since we cannot guarantee that the LD and birefringent principal axis of the entire composite system remain unchanged during the adjustment process, we consider a more general case. The principal axis of LD has an angle ${\theta }_{{{LD}}}$ with the *x*-axis, and the birefringent principal axis has an angle ${\phi }_{{{B}}}$ with the *x*-axis (we take the forward propagation case as an example for study). Then, the forward propagation permittivity tensor can be expressed as follows (which corresponds to the coordinates before rotation):

29 $${\varepsilon }^{{{{f}}}_{4}}=\left(\begin{array}{ccc}{\varepsilon }_{0}+{{i}}\gamma \cos 2{\theta }_{{{LD}}}-\rho \cos 2{\phi }_{{{B}}} & \rho \sin 2{\phi }_{{{B}}}-{{i}}\gamma \sin 2{\theta }_{{{LD}}}-{{i}}\mu +\chi & 0\\ \rho \sin 2{\phi }_{{{B}}}-{{i}}\gamma \sin 2{\theta }_{{{LD}}}+{{i}}\mu -\chi & {\varepsilon }_{0}-{{i}}\gamma \cos 2{\theta }_{{{LD}}}+\rho \cos 2{\phi }_{{{B}}} & 0\\ 0 & 0 & {\varepsilon }_{0}\end{array}\right)$$

For a plane wave propagating in the *z* direction (Forward), the permittivity tensor vector can be expressed as

30 $${\varepsilon }^{{{{f}}}_{4}}=\left(\begin{array}{cc}{\varepsilon }_{0}+{{i}}\gamma \cos 2{\theta }_{{{LD}}}-\rho \cos 2{\phi }_{{{B}}} & \rho \sin 2{\phi }_{{{B}}}-{{i}}\gamma \sin 2{\theta }_{{{LD}}}-{{i}}\mu +\chi \\ \rho \sin 2{\phi }_{{{B}}}-{{i}}\gamma \sin 2{\theta }_{{{LD}}}+{{i}}\mu -\chi & {\varepsilon }_{0}-{{i}}\gamma \cos 2{\theta }_{{{LD}}}+\rho \cos 2{\phi }_{{{B}}}\end{array}\right)$$

The corresponding eigenvalues and eigenstates are as follows:

31 $$\begin{array}{c}{\varepsilon }_{\,\,\,1,2}^{{{{f}}}_{4}}={\varepsilon}_{\,0}\pm {\lambda }_{{pt}}^{4}\\ |{\psi }_{\quad\,\,\pm }^{{{R,{f}}}_{4}}\rangle =\left[\begin{array}{c}1\\ \displaystyle\frac{({{i}}\gamma\cos 2{\theta }_{{{LD}}}-\rho\cos 2{\phi }_{{{B}}})\mp {\lambda }_{{pt}}^{4}}{-\left[(\rho \sin 2{\phi }_{{{B}}}-{{i}}\gamma \sin 2{\theta }_{{{LD}}})-{{i}}\mu +\chi \right]}\end{array}\right]\end{array}$$

where ${\lambda }_{{{pt}}}^{4}=\sqrt{{(\rho \cos 2{\phi }_{{{B}}}-{{i}}\gamma \cos 2{\theta }_{{{LD}}})}^{2}+{(\rho \sin 2{\phi }_{{{B}}}-{{i}}\gamma \sin 2{\theta }_{{{LD}}})}^{2}-{(\chi -{{i}}\mu )}^{2}}$. From the above, we can also derive the Jones matrix for Eq. (30):

32 $$\begin{array}{c}{t}^{{{{f}}}_{4}}=\left(\begin{array}{cc}{t}_{xx}^{{{{f}}}_{4}} & {t}_{xy}^{{{{f}}}_{4}}\\ {t}_{yx}^{{{{f}}}_{4}} & {t}_{yy}^{{{{f}}}_{4}}\end{array}\right)\\ =\displaystyle\frac{1}{2}\left[\begin{array}{cc}{\varphi }_{1}+{\varphi }_{2}-\displaystyle\frac{(\rho\cos 2{\phi }_{{{B}}}-i\gamma\cos 2{\theta }_{{{LD}}})}{{\lambda }_{{{pt}}}^{4}}({\varphi }_{1}-{\varphi }_{2}) & \displaystyle\frac{[\rho \sin 2{\phi }_{{{B}}}-i\gamma \sin 2{\theta }_{{{LD}}}+(\chi -{{i}}u)]}{{\lambda }_{{ {pt}}}^{4}}({\varphi }_{1}-{\varphi }_{2})\\ \displaystyle\frac{[\rho \sin 2{\phi }_{{{B}}}-{{i}}\gamma \sin 2{\theta }_{{{LD}}}-(\chi -{{i}}u)]}{{\lambda }_{{{pt}}}^{4}}({\varphi }_{1}-{\varphi }_{2}) & {\varphi }_{1}+{\varphi }_{2}+\displaystyle\frac{(\rho \cos 2{\phi }_{{{B}}}-{{i}}\gamma \cos 2{\theta }_{{{LD}}})}{{\lambda }_{{{pt}}}^{4}}({\varphi }_{1}-{\varphi }_{2})\end{array}\right]\end{array}$$

We then rotate the coordinates 45° clockwise. Consequently, Eq. (32) can be rewritten as follows:

33 $$\begin{array}{c}{t}^{{{{f}}^{\prime} }_{4}}=\left(\begin{array}{cc}{t}_{x^{\prime} x^{\prime} }^{{{{f}}^{\prime} }_{4}} & {t}_{x^{\prime} y^{\prime} }^{{{{f}}^{\prime} }_{4}}\\ {t}_{y^{\prime} x^{\prime} }^{{{{f}}^{\prime} }_{4}} & {t}_{y^{\prime} y^{\prime} }^{{{{f}}^{\prime} }_{4}}\end{array}\right)\\ =\displaystyle\frac{1}{2}\left[\begin{array}{cc}({\varphi }_{1}+{\varphi }_{2})+\displaystyle\frac{\rho \sin 2{\phi }_{{{B}}}-{{i}}\gamma \sin 2{\theta }_{{{LD}}}}{{\lambda }_{{{pt}}}^{4}}({\varphi }_{1}-{\varphi }_{2}) & \displaystyle\frac{[(\rho \cos 2{\phi }_{{{B}}}-{{i}}\gamma\cos 2{\theta }_{{{LD}}})+(\chi -{{i}}u)]}{{\lambda }_{{{pt}}}^{4}}({\varphi }_{1}-{\varphi }_{2})\\ \displaystyle\frac{[(\rho \cos 2{\phi }_{{{B}}}-{{i}}\gamma \cos 2{\theta }_{{{LD}}})-(\chi -{{i}}u)\left.\right)]}{{\lambda }_{{{pt}}}^{4}}({\varphi }_{1}-{\varphi }_{2}) & {\varphi }_{1}+{\varphi }_{2}-\displaystyle\frac{\rho \sin 2{\phi }_{{{B}}}-{{i}}\gamma \sin 2{\theta }_{{{LD}}}}{{\lambda }_{{{pt}}}^{4}}({\varphi }_{1}-{\varphi }_{2})\end{array}\right]\end{array}$$

To simplify the research process, we define three parameters $\Delta m$, $\Delta {h}_{1}$ and $\Delta {h}_{2}$, and construct a three-dimensional parameter space with these three parameters as dimensions. We also define another important parameter $g$, which is related to different Eps properties. Here

34 $$\begin{array}{c}\Delta {h}_{1}={\mathrm{Re}}\left\{{t}_{x^{\prime} x^{\prime} }^{{{{f}}^{\prime} }_{4}}-{t}_{y^{\prime} y^{\prime} }^{{{{f}}^{\prime} }_{4}}\right\}={\mathrm{Re}}\left\{\displaystyle\frac{\rho \sin 2{\phi }_{{{B}}}-{{i}}\gamma \sin 2{\theta }_{{{LD}}}}{{\lambda }_{{{pt}}}^{4}}({\varphi }_{1}-{\varphi }_{2})\right\}\\ \Delta {h}_{2}={\rm{Im}}\left\{{t}_{x^{\prime} x^{\prime} }^{{{{f}}^{\prime} }_{4}}-{t}_{y^{\prime} y^{\prime} }^{{{{f}}^{\prime} }_{4}}\right\}={\text{Im}}\left\{\displaystyle\frac{\rho \sin 2{\phi }_{{{B}}}-{{i}}\gamma\sin 2{\theta }_{{{LD}}}}{{\lambda }_{{{pt}}}^{4}}({\varphi }_{1}-{\varphi }_{2})\right\}\\ \Delta m=\left|{t}_{y^{\prime} x^{\prime} }^{{{{f}}^{\prime} }_{4}}\right|=\left|\displaystyle\frac{1}{2} \frac{[(\rho \cos 2{\phi }_{{{B}}}-{{i}}\gamma\cos 2{\theta }_{{{LD}}})-(\chi -{{i}}u)\left.\right)]}{{\lambda }_{{{pt}}}^{4}}({\varphi }_{1}-{\varphi }_{2})\right|\\ g={t}_{x^{\prime} y^{\prime} }^{{{{f}}^{\prime} }_{4}}=\displaystyle\frac{1}{2} \frac{[(\rho \,\cos 2{\phi }_{{{B}}}-{{i}}\gamma \,\cos 2{\theta }_{{{LD}}})+(\chi -{{i}}u)]}{{\lambda }_{{{pt}}}^{4}}({\varphi }_{1}-{\varphi }_{2})\end{array}$$

We find that for the composite non-Hermitian system to reach the EP and have a degenerate eigenstate of ${(1,1)}^{{{T}}}$, the Jones matrix must conform to the form presented in Eq. (27). Then it is necessary to satisfy $\Delta m=0,\Delta {h}_{1}=\Delta {h}_{2}=0,g\,\ne \,0$. When $\Delta {h}_{1}=\Delta {h}_{2}=0$, it means ${\theta }_{LD}={\phi }_{B}=0$. At this time, the LD and birefringent principal axis of the system are (1, 0)−(0, 1) axis ((1’, 1’)−(1’, −1’) axis). Thus, the forward propagation permittivity tensor returns to the situation described in Eq. (1) of the above theoretical study. Our composite system is back to the general case (the principal axes of both LD and birefringence are in the directions of *x* and *y*-axis). On this basis, $\Delta m=0$, that is $\rho =\chi ,\gamma =\mu$, which is consistent with one of the conditions for the occurrence of EP in theoretical Eq. (1). In addition, for the entire composite system to achieve an EP, it is necessary to satisfy $g\ne 0$, which is easily obtainable. When the composite system is at the EP, $g=- {i}(\chi - {i}\mu )\frac{2\pi d{{\varepsilon }_{0}}^{-\frac{1}{2}}}{{\varLambda }_{0}}{\varphi }_{0}$. It mainly affects the properties (speed, trajectory) of state evolution. Here, we observe that while the EPs share identical degenerate eigenvalue and eigenstate, due to different *C* ($\gamma /\chi =C=\mu /\rho$) values, the *g* values are different, resulting in different evolution trajectories for these EPs. However, no matter what *C* value and *g* value ($g\ne 0$), as long as we adjust the parameters to $\Delta m=\Delta {h}_{1}=\Delta {h}_{2}=0$, we will adjust the non-Hermitian system to the EP with degenerate eigenstates of ${(1,1)}^{{{T}}}$. In addition, considering that the loss present in the experiment will affect the values of the above three parameters ($\Delta m,\Delta {h}_{1},\Delta {h}_{2}$), and thus influence our judgment on the state near the EP. We then reduce Eq. (33), which can be rewritten as follows:

35 $$\begin{array}{c}{t}^{{{{f}}^{\prime} }_{4}}=\left(\begin{array}{cc}{t}_{x^{\prime} x^{\prime} }^{{{{f}}^{\prime} }_{4}} & {t}_{x^{\prime} y^{\prime} }^{{{{f}}^{\prime} }_{4}}\\ {t}_{y^{\prime} x^{\prime} }^{{{{f}}^{\prime} }_{4}} & {t}_{y^{\prime} y^{\prime} }^{{{{f}}^{\prime} }_{4}}\end{array}\right)\\ ={t}_{x^{\prime} x^{\prime} }^{{{{f}}^{\prime} }_{4}}\left(\begin{array}{cc}1 & \displaystyle\frac{{t}_{x^{\prime} y^{\prime} }^{{{{f}}^{\prime} }_{4}}}{{t}_{x^{\prime} x^{\prime} }^{{{{f}}^{\prime}}_{4}}}\\ \displaystyle\frac{{t}_{y^{\prime} x^{\prime} }^{{{{f}}^{\prime} }_{4}}}{{t}_{x^{\prime} x^{\prime} }^{{f^{\prime} }_{4}}} & \displaystyle\frac{{t}_{y^{\prime} y^{\prime} }^{{{{f}}^{\prime} }_{4}}}{{t}_{x^{\prime} x^{\prime} }^{{{{f}}^{\prime} }_{4}}}\end{array}\right)\\ ={t}_{x^{\prime} x^{\prime} }^{{{{f}}^{\prime} }_{4}}\left(\begin{array}{cc}1 & \displaystyle\frac{{t}_{x^{\prime} y^{\prime} }^{{{{f}}^{\prime} }_{4}}}{{t}_{x^{\prime} x^{\prime} }^{{{{f}}^{\prime}}_{4}}}\\ \displaystyle\frac{{t}_{y^{\prime} x^{\prime} }^{{{{f}}^{\prime} }_{4}}}{{t}_{x^{\prime} x^{\prime} }^{{{{f}}^{\prime}}_{4}}} & 1-\displaystyle\frac{{t}_{x^{\prime} x^{\prime} }^{{{{f}}^{\prime} }_{4}}-{t}_{y^{\prime} y^{\prime} }^{{{ {f}}^{\prime} }_{4}}}{{t}_{x^{\prime} x^{\prime} }^{{{{f}}^{\prime} }_{4}}}\end{array}\right)\end{array}$$

Similarly, Eq. (34) can be rewritten as follows:

36 $$\begin{array}{l} \Delta {h^{\prime}_{1} }={\mathrm{Re}}\left\{-\displaystyle\frac{{t}_{x^{\prime} x^{\prime} }^{{{{f}}^{\prime} }_{4}}-{t}_{y^{\prime} y^{\prime} }^{{f^{\prime} }_{4}}}{{t}_{x^{\prime} x^{\prime} }^{{{{f}}^{\prime} }_{4}}}\right\}={\mathrm{Re}}\left\{-\displaystyle\frac{\displaystyle\frac{\rho \sin 2{\phi }_{{{B}}}-{{i}}\gamma \sin 2{\theta }_{{LD}}}{{\lambda }_{{{pt}}}^{4}}({\varphi }_{1}-{\varphi }_{2})}{\left[({\varphi }_{1}+{\varphi }_{2})+\displaystyle\frac{\rho \sin 2{\phi }_{{{B}}}-{{i}}\gamma \sin 2{\theta }_{{{LD}}}}{{\lambda }_{{{pt}}}^{4}}({\varphi }_{1}-{\varphi }_{2})\right]}\right\} \\ \Delta {h^{\prime}_{2} }={\rm{Im}}\left\{-\displaystyle\frac{{t}_{x^{\prime} x^{\prime} }^{{{{f}}^{\prime} }_{4}}-{t}_{y^{\prime} y^{\prime} }^{{{{f}}^{\prime} }_{4}}}{{t}_{x^{\prime} x^{\prime} }^{{{{f}}^{\prime} }_{4}}}\right\}={\rm{Im}}\left\{-\displaystyle\frac{\displaystyle\frac{\rho \sin 2{\phi }_{{{B}}}-{{i}}\gamma \sin 2{\theta }_{{{LD}}}}{{\lambda }_{{{pt}}}^{4}}({\varphi }_{1}-{\varphi }_{2})}{\left[({\varphi }_{1}+{\varphi }_{2})+\displaystyle\frac{\rho \sin 2{\phi }_{{{B}}}-{{i}}\gamma \sin 2{\theta }_{{{LD}}}}{{\lambda }_{{ {pt}}}^{4}}({\varphi }_{1}-{\varphi }_{2})\right]}\right\} \\ \Delta m^{\prime} =\left\{\begin{array}{c}\left|\displaystyle\frac{{t}_{y^{\prime} x^{\prime} }^{{{{f}}^{\prime} }_{4}}}{{t}_{x^{\prime} x^{\prime} }^{{{ {f}}^{\prime} }_{4}}}\right|=\left|\displaystyle\frac{1}{2}\frac{[(\rho \cos 2{\phi }_{{{B}}}-{{i}}\gamma \cos 2{\theta }_{{{LD}}})-(\chi -{{i}}u)\left.\right)] ({\varphi }_{1}-{\varphi }_{2})}{{\lambda }_{{{pt}}}^{4}\left[({\varphi }_{1}+{\varphi }_{2})+\displaystyle\frac{\rho \sin 2{\phi }_{{{B}}}-{{i}}\gamma \sin 2{\theta }_{{{LD}}}}{{\lambda }_{{{pt}}}^{4}}({\varphi }_{1}-{\varphi }_{2})\right]}\right|\cdots \cdots {\rm {Arg}}\left(\displaystyle\frac{{t}_{y^{\prime} x^{\prime} }^{{{{f}}^{\prime} }_{4}}}{{t}_{x^{\prime} x^{\prime} }^{{{{f}}^{\prime} }_{4}}}\right)\, > \,0\\ -\left|\displaystyle\frac{{t}_{y^{\prime} x^{\prime} }^{{{{f}}^{\prime} }_{4}}}{{t}_{x^{\prime} x^{\prime} }^{{{{f}}^{\prime} }_{4}}}\right|=-\left|\displaystyle\frac{1}{2} \frac{[(\rho \cos 2{\phi }_{{{B}}}-{{i}}\gamma \cos 2{\theta }_{{{LD}}})-(\chi -{{i}}u)\left.\right)] ({\varphi }_{1}-{\varphi }_{2})}{{\lambda }_{{{pt}}}^{4} \left[({\varphi }_{1}+{\varphi }_{2})+\displaystyle\frac{\rho \sin 2{\phi }_{{{B}}}-{{i}}\gamma \sin 2{\theta }_{{{LD}}}}{{\lambda }_{{{pt}}}^{4}}({\varphi }_{1}-{\varphi }_{2})\right]}\right|\cdots \cdots {\rm {Arg}}\left(\displaystyle\frac{{t}_{y^{\prime} x^{\prime} }^{{{{f}}^{\prime} }_{4}}}{{t}_{x^{\prime} x^{\prime} }^{{{{f}}^{\prime} }_{4}}}\right)\le 0\end{array}\right. \\ g^{\prime} =\displaystyle\frac{{t}_{x^{\prime} y^{\prime} }^{{{{f}}^{\prime} }_{4}}}{{t}_{x^{\prime} x^{\prime} }^{{{{f}}^{\prime} }_{4}}}=\displaystyle\frac{1}{2} \frac{[(\rho \cos 2{\phi }_{{{B}}}-{{i}}\gamma \cos 2{\theta }_{{{LD}}})+(\chi -{{i}}u)] ({\varphi }_{1}-{\varphi }_{2})}{{\lambda }_{{{pt}}}^{4} \left[({\varphi }_{1}+{\varphi }_{2})+\displaystyle\frac{\rho \sin 2{\phi }_{{{B}}}-{{i}}\gamma \sin 2{\theta }_{{{LD}}}}{{\lambda }_{{{pt}}}^{4}}({\varphi }_{1}-{\varphi }_{2})\right]} \end{array}$$

where the value range of ${\rm {Arg}}\left(\frac{{t}_{y^{\prime} x^{\prime} }^{{{{f}}^{\prime} }_{4}}}{{t}_{x^{\prime} x^{\prime} }^{{{{f}}^{\prime} }_{4}}}\right)$ is from −*π* to *π*. Here, when the radiation angle of $\Delta m^{\prime}$ is greater than zero, its value is its modulus. When the radiation angle is less than or equal to zero, take the opposite number of the modulus.

The new four parameters ($\Delta m^{\prime} ,\Delta {h^{\prime}_{1} },\Delta {h^{\prime}_{2} },g^{\prime}$) are directly related to the previous four parameters ($\Delta m,\Delta {h}_{1},\Delta {h}_{2},g$). Similar to the above description, by adjusting the three-dimensional coordinate point ($\Delta m^{\prime} ,\Delta {h^{\prime}_{1} },\Delta {h^{\prime}_{2} }$) to (0, 0, 0), we can also adjust the non-Hermitian system to the EP with a degenerate eigenstate of ${(1,1)}^{{{T}}}$.

Next, we discuss how to adjust the angle ${\theta }_{{{D}}i}$ of the dichroic material, the angle ${\theta }_{{{Bib}}}$ of the Babinet compensator, the loss coefficient ${\alpha }_{{{D}}i}$ of the dichroic material, and the birefringence coefficient ${\beta }_{{{Bib}}}$ of the Babinet compensator to modulate the 3D coordinate point ($\Delta m^{\prime} ,\Delta {h^{\prime}_{1} },\Delta {h^{\prime}_{2} }$) to (0, 0, 0).

Firstly, the transmission characteristics of the composite experimental system are discussed theoretically. For a composite system consisting of a large area extrinsic chiral metasurface, a dichroic material and a Babinet compensator, the Jones matrix form with *x* and *y* as base vectors can be given as follows:

37 $${t}^{{{f}},\exp }={t}^{{{f}},{{Bib}}}\ast {t}^{{{f}},{{D}}i}\ast {t}^{{{f}},{{M}}}$$

where the Jones matrix of the dichroic material is

38 $${t}^{{{f,D}}i}={\left(\begin{array}{cc}\cos {\theta }_{{{D}}i} & -\sin {\theta }_{{{D}}i}\\ \sin {\theta }_{{{D}}i} & \cos {\theta }_{{{D}}i}\end{array}\right)}^{-1}\ast \left(\begin{array}{cc}1 & 0\\ 0 & { {{e}}}^{-{\alpha }_{{{D}}i}}\end{array}\right)\ast \left(\begin{array}{cc}\cos {\theta }_{{{D}}i} & -\sin {\theta }_{{{D}}i}\\ \sin {\theta }_{{{D}}i} & \cos {\theta }_{{{D}}i}\end{array}\right)$$

The Jones matrix of the Babinet compensator is

39 $${t}^{{{f}},{{Bib}}}={\left(\begin{array}{cc}\cos {\theta }_{{{Bib}}} & -\sin {\theta }_{{{Bib}}}\\ \sin {\theta }_{{{Bib}}} & \cos {\theta }_{{{Bib}}}\end{array}\right)}^{-1}\ast \left(\begin{array}{cc}1 & 0\\ 0 & {{{e}}}^{-{{i}}{\beta }_{{{Bib}}}}\end{array}\right)\ast \left(\begin{array}{cc}\cos {\theta }_{{{Bib}}} & -\sin {\theta }_{{{Bib}}}\\ \sin {\theta }_{{{Bib}}} & \cos {\theta }_{{{Bib}}}\end{array}\right)$$

The Jones matrix of the metasurface is

40 $$\displaystyle{t}^{{{f,M}}}=\left(\begin{array}{cc}{t}_{xx}^{{{f,M}}} & {t}_{xy}^{{{f,M}}}\\ {t}_{yx}^{{{f,M}}} & {t}_{yy}^{{{f,M}}}\end{array}\right)$$

Out of an intuitive expression, we transform the coordinate axis 45° clockwise around the *z*-axis, and the Jones matrix of the above composite system can be rewritten as

41 $${t}^{{{f}}^{\prime} ,\exp }=\frac{1}{\sqrt{2}}{\left(\begin{array}{cc}1 & -1\\ 1 & 1\end{array}\right)}^{-1}\ast {t}^{{{f}},{{Bib}}}\ast {t}^{{{f,D}}i}\ast {t}^{{{f,M}}}\ast \frac{1}{\sqrt{2}}\left(\begin{array}{cc}1 & -1\\ 1 & 1\end{array}\right)$$

From the above, it can be known that to make the Jones matrix of the experimental composite system satisfy the form when the system is at the EP, we need to adjust the four parameters of ${\theta }_{{{D}}i}$, ${\theta }_{{{Bib}}}$, ${\alpha }_{{{D}}i}$, ${\beta }_{{{Bib}}}$ in the experiment, such that $\varDelta m^{\prime} =\varDelta {h^{\prime}_{1} }=\varDelta {h^{\prime}_{2} }=0$, which means the 3D coordinate point ($\varDelta m^{\prime} ,\varDelta {h^{\prime}_{1} },\varDelta {h^{\prime}_{2} }$) reach the (0, 0, 0).

**Fig. 5:**
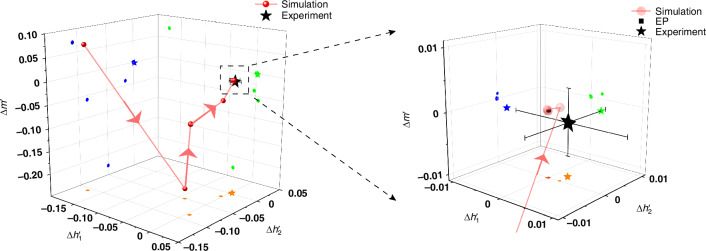
The three-dimensional coordinate points are obtained by adjusting four parameters experimentally and simulatively. Where the black square represents the position of the EP’s 3D coordinate point. The black five-pointed star represents the final 3D coordinate point obtained by the experiment after several cycles of adjustment. The red balls denote the multiple 3D coordinate points obtained from the simulation calculation, while the arrows illustrate the evolution process of the composite system from a state far away from an EP to near an EP by adjusting the four parameters. The inset is a magnified area around EP’s 3D coordinate point

During the experiment, the Jones matrix of the entire non-Hermitian system can be measured. The measurement method is described in S6 of the supplementary materials. Thus, we are able to obtain and display the 3D coordinate point on the computer through calculation. In the adjustment process of the experiment, we adopted the method of cyclically adjusting four parameters one by one, aiming to accurately regulate the composite system to reach a near EP. First, adjust one of the four parameters and fix the remaining three parameters, adjust the 3D coordinate point to the position closest to (0, 0, 0). Then, we fix the first already adjusted parameter and adjust one of the remaining three parameters, and repeat the previous steps. After completing the adjustment of the four controllable parameters separately, enter the next cycle adjustment process. In the experiment, after several rounds of adjustment, we adjusted the non-Hermitian system to be near the EP. The 3D coordinate point of the final near-EP obtained in the experiment is shown in Fig. 5 (black five-pointed star). In order to more vividly illustrate our experimental adjustment process, we present it through computational simulation. In this computational simulation, we replace the adjustment process of the actual experiment by taking points and simulate the experiment process by calculating the 3D coordinate points of each taking point (specific steps are described in S7 of the supplementary material). The evolution of 3D coordinate points is illustrated in Fig. 5 (each red ball represents a 3D coordinate point after a cyclic taking of points), where the orange, blue, and green dots are the projections of 3D coordinate points in three dimensions. After several cycles of taking points process of the four parameters, the 3D coordinate points can reach a situation very close to EP from far away from that. The inset in Fig. 5 more clearly demonstrates the situation where the final three-dimensional coordinate points obtained through our experiments are after adjustment. In the future, by developing a real-time measurement and control system, we can achieve real-time adjustment. Moreover, incorporating more sophisticated adjustment elements enables precise adjustment closer to the EPs.

In addition, during both experimental and numerical simulation processes, we observe that varying initial Jones matrices and adjustment processes drive the 3D chiral non-Hermitian composite system to approach different EPs. Despite this, these EPs share identical degenerate eigenstates and eigenvalues, while exhibiting distinct values of $g^{\prime}$. Consequently, this results in different evolution trajectories of the polarization states. The relevant results are shown in S8 of the supplementary materials. This result demonstrates the broad applicability of our proposed method. Furthermore, this is also consistent with our theory above, that is, for any value of *C*, the system can reach an EP of ${(1,1)}^{T}$. In the experiment, we adjusted the system to approach an EP with a very large *C* value, whose evolution curve is similar to that when $C=\infty$.

**Fig. 6:**
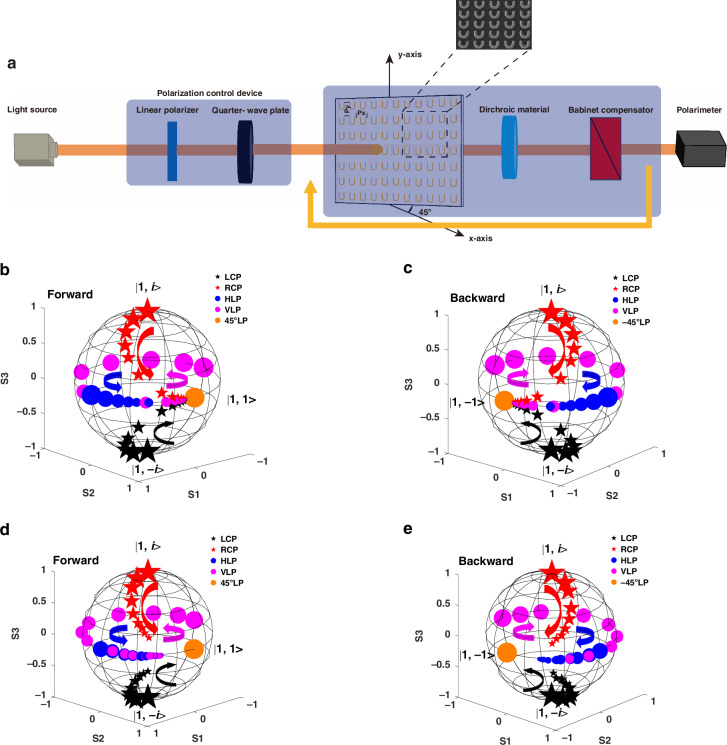
The experimental setup of the 3D chiral non-Hermitian system and the polarization state evolution of the transmitted light at a near-EP state. **a** Experimental setup for the realization of the proposed system. The inset is an SEM image of the extrinsic chiral metasurface, and the yellow trace represents the cyclic measurement of the transmitted polarization. **b** and **c** Poincaré sphere tracks of polarization evolution as several selected input states passing through the proposed 3D chiral non-Hermitian system are calculated at the EP for the forward and backward directions, and the direction of the evolution trajectory is indicated by arrows. **d** and **e** Poincaré spheres describe the experimental polarization evolution of several selected input polarization states obliquely incident on the extrinsic chiral metasurface from the forward and backward directions at a wavelength of 1550 nm, respectively. Here, the five-pointed star symbol represents circularly polarized light, red represents right-polarized light, black represents left-polarized light, and the circular symbol represents linearly polarized light, where blue and purple represent the HLP and VLP, respectively. The orange are 45° LP for forward propagation and −45° LP for backward propagation

Next, we experimentally measured the evolution of the polarization state of the transmitted light in the 3D chiral non-Hermitian system. To achieve this purpose, the transmitted polarization state is taken as the incident polarization state of the subsequent measurement, and the polarization evolution process of an incident polarization state can be obtained by repeating this measurement. We select horizontal linear polarization (HLP), vertical linear polarization (VLP), left circular polarization (LCP), and right circular polarization (RCP) as the input polarization states. The evolutions of the polarization states are recorded on the Poincaré sphere, as shown in Fig. 6d, e (which correspond to the coordinates before rotation). As shown in Fig. 6d, for forward propagation, regardless of the linear polarization incidence or circular polarization incidence, the polarization evolution trend is finally toward approximately 45° LP. However, as the polarization state of evolution approaches the eigenstate, the rate of evolution decreases, making it challenging to discern the evolution using a polarimeter. Consequently, the evolution of adjacent eigenstates is not measured. Similarly, for backward incidence, regardless of the incident polarization state, it will eventually evolve to approximately −45°LP, as shown in Fig. 6e. The experimental results are in good agreement with the calculated results obtained from the forward and backward Jones matrices, as shown in Fig. 6b and c. The small differences between the experimental and calculated results indicate that the experimental system is close to $C=\infty$ and achieves a near EP rather than an exact EP. These results further prove that the Jones matrix of our experimental system approximately satisfies Eqs. (27) and (28), and that the system is in a state close to EP. In addition, in this way, we experimentally prove that asymmetric state switching can be achieved in 3D chiral non-Hermitian optical systems because of the difference in Jones matrices during forward and backward propagation. An omni-polarizer can be realized at the EP. Thus, both experiments and numerical calculations demonstrate the feasibility of the proposed method, offering a novel approach to studying more abundant non-Hermitian systems.

## Discussion

In conclusion, we demonstrate theoretically, simulatively, and experimentally that non-Hermitian systems based on 3D chirality can be employed to achieve asymmetric mode/state switching and omni-polarizer action at the EP without the encircling process. We propose a 3D chiral non-Hermitian system by introducing the permittivity tensor of a 3D chiral material into the non-Hermitian system. The eigenstate evolution of the system with the change of $\left|\frac{\gamma }{\mu }\right|$ and the evolution of light polarization states for several systems with a specific $\left|\frac{\gamma }{\mu }\right|$ are analyzed during forward and backward propagation when $C=1$. Our results show that due to the different permittivity tensor matrices and Jones matrices of 3D chiral non-Hermitian systems during forward and backward propagation, the eigenstates are different, which results in asymmetric state switching. In particular, at the EP of a 3D chiral non-Hermitian system, omni-polarizer action can be realized. More importantly, we demonstrate that 3D chiral non-Hermitian systems can be tuned to a state near the EP using a dichroic material and a Babinet compensator. Utilizing this method, a simple free-space 3D chiral non-Hermitian system is constructed and adjusted to a state near an EP. Then, the evolution behavior of the light polarization state near the EP is directly observed, which is quite consistent with our theory. The experiment proves that a 3D chiral non-Hermitian system can realize asymmetric state switching and omni-polarizer action at the EP. Our work provides a new perspective for the study of non-Hermitian systems and provides a theoretical and experimental basis for the application of 3D chiral non-Hermitian systems, such as non-Hermitian-enhanced chirality detection and ultra-sensitive chiral polarization manipulation.

## Materials and methods

### Fabrications

The extrinsic chiral metasurface utilized in this study comprises a rectangular lattice array of gold split-ring resonators (SRRs) supported by a glass substrate. The gold split-ring resonators were fabricated using electron beam lithography (EBL).

## Supplementary information

Supplemental material

## Data Availability

The data that support the findings of this study are available from the corresponding author upon reasonable request.
