# Supplementary material for: Non-Hermitian systems based on 3D chirality enabled asymmetrical polarization switching and omni-polarizer action at an EP
Source: Light Sci Appl. 2025 Nov 18;14:383. doi: 10.1038/s41377-025-01960-5 (PMC12627468; doi:10.1038/s41377-025-01960-5)
Supplement: Supplementary file 1 — Supplemental material [file 41377_2025_1960_MOESM1_ESM.docx]

**Supporting Information for**

**Non-Hermitian systems based on 3D chirality enabled asymmetrical polarization switching and omni-polarizer action at an EP**

Xianhui Fu,^1†^ Hao Hu,^1†^ Jiawei Zhang,^1†^ Jiwei Qi,^1,*^ Sihao Zhang,^1^ Qiang Wu,^1^ Yao Lu,^1^ Zongqiang Chen,^1^ Jing Chen,^1^ Xuanyi Yu,^1^ Qian Sun,^1,*^ Jingjun Xu^1,*^

^1^ Key Laboratory of Weak-Light Nonlinear Photonics, Ministry of Education, Tianjin Key Laboratory of Photonics and Technology of Information Science, TEDA Institute of Applied Physics and School of Physics, Nankai University, Tianjin 300457, China

^*^e-mail: qijw@nankai.edu.cn

^*^e-mail: qiansun@nankai.edu.cn

^*^e-mail: [jjxu@nankai.edu.cn](mailto:jjxu@nankai.edu.cn)

^†^These authors contributed equally to this work.

**S1: The evolution of eigenstates with the change of parameters and the evolution of polarization states under certain parameters for two special cases of 3D non-Hermitian systems**

We discuss in detail two special cases of 3D non-Hermitian systems in general, one contains only linear dichroism (LD) and chiral optical rotation without birefringence and CD. The forward and backward permittivity-tensors of the system are as follows [1]:

$\varepsilon^{f_{2}}=\left(\begin{matrix} \varepsilon_{0}+i\gamma& -i\mu& 0 \\ i\mu& \varepsilon_{0}-i\gamma& 0 \\ 0 & 0 & \varepsilon_{0} \end{matrix}\right)$ and $\varepsilon^{b_{2}}=\left(\begin{matrix} \varepsilon_{0}+i\gamma& i\mu& 0 \\ -i\mu& \varepsilon_{0}-i\gamma& 0 \\ 0 & 0 & \varepsilon_{0} \end{matrix}\right)$

Where $\varepsilon_{0}$ is a positive real number, *γ* and *μ* represent the amount of gain or loss and coupling (chiral optical rotation), respectively (*x*: gain axis, *y*: loss axis). For a plane wave propagating in the *z* direction (Forward), the permittivity-tensors can be expressed as

$$\varepsilon^{f_{2}}=\left(\begin{matrix} \varepsilon_{0}+i\gamma& -i\mu\\ i\mu& \varepsilon_{0}-i\gamma\end{matrix}\right)$$

Then, two eigenvalues can be solved:

$$\varepsilon^{f_{2}}_{1,2}=\varepsilon_{0}\pm\lambda''_{pt}$$

Where$\lambda''_{pt}=\sqrt{\mu^{2}-\gamma^{2}}$, and the corresponding two eigenstates are as follows:

$$|\psi^{R,f_{2}}_{\pm}\rangle=\left(\begin{matrix} 1 \\ \frac{\gamma}{\mu}\pm i\sqrt{1-\left(\frac{\gamma}{\mu}\right)^{2}} \end{matrix}\right)$$

When light propagates in the *-z* direction (backward), the reciprocity theorem is applied, and the form of the permittivity- tensor is as follows:

$$\varepsilon^{b_{2}}=\left(\begin{matrix} \varepsilon_{0}+i\gamma& i\mu\\ -i\mu& \varepsilon_{0}-i\gamma\end{matrix}\right)$$

The corresponding eigenvalues and eigenstates are as follows:

$$\varepsilon^{b_{2}}_{1,2}=\varepsilon{}_{0}\pm\lambda''_{pt}$$

$$|\psi^{R,b_{2}}_{\pm}\rangle=\left(\begin{matrix} 1 \\ -\frac{\gamma}{\mu}\mp i\sqrt{1-\left(\frac{\gamma}{\mu}\right)^{2}} \end{matrix}\right)$$

Here, the superscript *f* denotes forward propagation, *R* denotes right eigenstates, and the superscript *b* denotes backward propagation. As can be seen from the Eq. (S3), Eq. (S4), Eq. (S5) and Eq. (S6), the eigenvalues for forward and backward propagation are the same, whereas the eigenstates are different. This characteristic distinguishes such systems from other non-Hermitian media.

Fig. S1(a) and (b) show the eigenstate evolution of the system $C=\infty$ during forward and backward propagation with the change of$\left|\frac{\gamma}{\mu}\right|$, respectively. When$\left|\frac{\gamma}{\mu}\right|=\infty(\gamma\neq0,\mu=0)$, the eigenstates of forward and backward propagation are $(1,0)^{T}$and $(0,1)^{T}$, where the gain eigenstate is $(1,0)^{T}$ and the loss eigenstate is $(0,1)^{T}$ [2]. When, with decreasing$\left|\frac{\gamma}{\mu}\right|$, the two eigenstates $|\psi_{\pm}^{R,f_{2}}\rangle$ converge from $(1,0)^{T}$ and $(0,1)^{T}$ to $(1,1)^{T}$, while $|\psi_{\pm}^{R,b_{2}}\rangle$ converges from $(1,0)^{T}$ and $(0,1)^{T}$ to $(1,-1)^{T}$, respectively, represented by the yellow trajectory on the sphere. When $\left|\frac{\gamma}{\mu}\right|=1$, the EP occurs in both the forward and backward propagation systems. That is, the eigenstates of forward and backward propagation degenerate at $(1,1)^{T}$ and$(1,-1)^{T}$, respectively. As $\left|\frac{\gamma}{\mu}\right|$ continues to decrease, when$\left|\frac{\gamma}{\mu}\right|<1$, the eigenstates $|\psi_{\pm}^{R,f_{2}}\rangle$ and $|\psi_{\pm}^{R,b_{2}}\rangle$ split from the $(1,1)^{T}$ and $(1,-1)^{T}$ to the $(1,i)^{T}$ and $(1,-i)^{T}$ states respectively, represented by the blue trajectory on the sphere. When $\left|\frac{\gamma}{\mu}\right|=0(\gamma=0,\mu\neq0)$, the eigenstates$|\psi_{\pm}^{R,f_{2}}\rangle$and$|\psi_{\pm}^{R,b_{2}}\rangle$are still different, that is,$|\psi_{\pm}^{R,f_{2}}\rangle=|\psi_{\mp}^{R,b_{2}}\rangle$. Hence, the eigenstate evolution process shows that the eigenstates of the proposed system are different for forward and backward propagation when chirality exists.


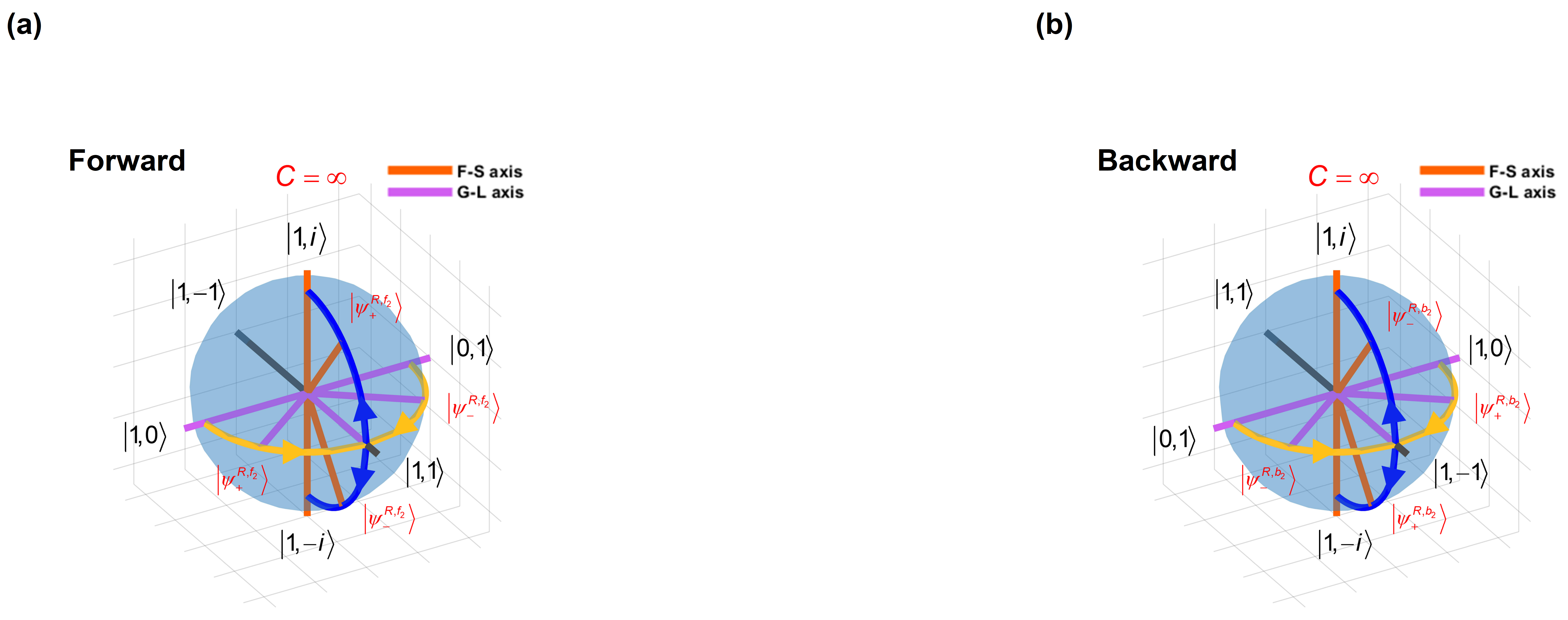


Fig. S1 Poincaré spheres showing the evolution of eigenstates of light passing through such a special 3D chirality non-Hermitian system with $C=\infty$ in both the forward (a) and backward (b) directions.

Then, for several systems with a specific $\left|\frac{\gamma}{\mu}\right|$, the evolution of light polarization states during forward and backward propagation is analyzed. When forward propagation is considered, in the broken phase of $\left|\frac{\gamma}{\mu}\right|=\frac{2\sqrt{3}}{3}$, the eigenvalues are two different complex numbers in Eq. (S3) and the corresponding eigenstates are two nonorthogonal linear polarizations that experience equal gain and loss. The polarization dynamics in this case are depicted on the Poincaré sphere, as shown in Fig. S2(a). Since $|\psi_{+}^{R,f_{2}}\rangle$ is the gain eigenstate, and $|\psi_{-}^{R,f_{2}}\rangle$is the loss eigenstate, any polarization eigenstate except the loss eigenstate $|\psi_{-}^{R,f_{2}}\rangle$ will be amplified and rotated toward the gain eigenstate $|\psi_{+}^{R,f_{2}}\rangle$, as described in the yellow and purple trajectories on the Poincaré sphere. Nevertheless, in the exact phase of $\left|\frac{\gamma}{\mu}\right|=\frac{1}{3}$, the eigenvalues are two different real numbers, and the corresponding eigenstates are two elliptic polarization states. The other polarization states precess in circles about nonorthogonal eigenstates $|\psi_{\pm}^{R,f_{2}}\rangle$**,** as shown in Fig. S2(b). For $\left|\frac{\gamma}{\mu}\right|=\frac{2\sqrt{3}}{3}$in the backward propagation direction, the result is similar to that of forward propagation, with the exception of the loss eigenstate$|\psi_{-}^{R,b_{2}}\rangle$, all polarization states are amplified toward the gain eigenstate $|\psi_{+}^{R,b_{2}}\rangle$. However, a notable distinction arises from the fact that the forward and backward polarization eigenstates in our system are different, so the gain eigenstate$|\psi_{+}^{R,b_{2}}\rangle$ and loss eigenstate $|\psi_{-}^{R,b_{2}}\rangle$ are different from those of forward propagation, resulting in an opposite direction of the evolution trajectory of the eigenstates compared with forward propagation, as shown in Fig. S2(d). Moreover, for $\left|\frac{\gamma}{\mu}\right|=\frac{1}{3}$ in backward propagation, the result is also analogous to that in forward propagation. The polarization state precesses in circles around a pair of nonorthogonal states$|\psi_{\pm}^{R,b_{2}}\rangle$. The yellow trajectory on the sphere in Fig. S2(e) is shown. Similarly, the direction of rotation of the polarization state is opposite to that of forward propagation. Eq. (S3) and Eq. (S6) indicate that when $\left|\frac{\gamma}{\mu}\right|=1$, EP occurs in both forward propagation and backward propagation, where the eigenstates coalesce into $(1,1)^{T}$ and $(1,-1)^{T}$ in the forward and backward directions respectively. And no matter what polarization state is incident, the polarization of light will evolve toward the eigenstate, as shown in Fig. S2(c) and Fig. S2(f). At this time, the polarization of light propagating forward and backward will evolve toward two vertically degenerate eigenstates. Therefore, we can conclude that due to the distinct eigenstates of forward and backward propagation of 3D chiral non-Hermitian systems with $C=\infty$ , polarized light will experience different polarization evolutions when transmitted forward and backward, resulting in asymmetric state switching. When the system is at the EP, the light propagating forward and backward will end up in two orthogonal polarization states after a sufficiently long evolutionary distance. On this basis, an omni-polarizer is realized.


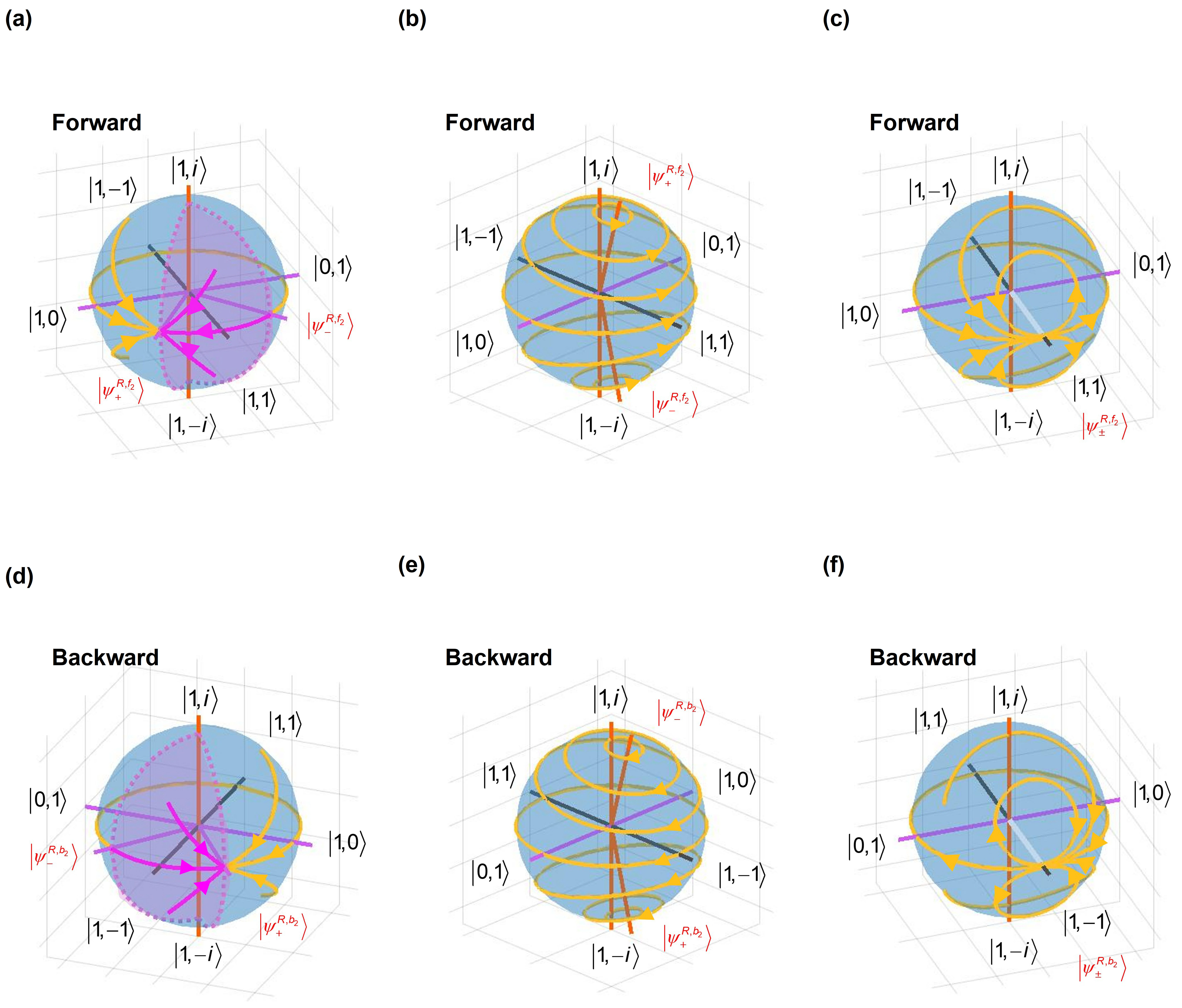


Fig. S2 Poincaré spheres describe the polarization dynamics of light passing through a special 3D chirality non-Hermitian system with $C=\infty$ in both the forward and backward directions for a specific $\left|\frac{\gamma}{\mu}\right|$, where (a), (d) $\left|\frac{\gamma}{\mu}\right|=\frac{2\sqrt{3}}{3}$, (b), (e) $\left|\frac{\gamma}{\mu}\right|=\frac{1}{3}$and (c), (f) $\left|\frac{\gamma}{\mu}\right|=1$.

The other special case contains only birefringence and CD without LD and chiral optical rotation. Similarly, the forward and backward permittivity- tensors of the system can be given:

$$\varepsilon^{f_{3}}=\left(\begin{matrix} \varepsilon_{0}-\rho& \chi\\ -\chi& \varepsilon_{0}+\rho\end{matrix}\right)$$

The corresponding eigenvalues and eigenstates are as follows:

$$\varepsilon^{f_{3}}_{1,2}=\varepsilon{}_{0}\pm\lambda'''_{pt}$$

$$|\psi^{R,f_{3}}_{\pm}\rangle=\left(\begin{matrix} 1 \\ \frac{\rho}{\chi}\pm i\sqrt{1-\left(\frac{\rho}{\chi}\right)^{2}} \end{matrix}\right)$$

where$\lambda'''_{pt}=\sqrt{\rho^{2}-\chi^{2}}$, and

$$\varepsilon^{b_{3}}=\left(\begin{matrix} \varepsilon_{0}-\rho& -\chi\\ \chi& \varepsilon_{0}+\rho\end{matrix}\right)$$

The corresponding eigenvalues and eigenstates are as follows:

$$\varepsilon^{b_{3}}_{1,2}=\varepsilon{}_{0}\pm\lambda'''_{pt}$$

$$|\psi^{R,b_{3}}_{\pm}\rangle=\left(\begin{matrix} 1 \\ -\frac{\rho}{\chi}\mp i\sqrt{1-\left(\frac{\rho}{\chi}\right)^{2}} \end{matrix}\right)$$

When $\rho=\chi$, EP occurs in both forward propagation and backward propagation, where the eigenstates coalesce into $(1,1)^{T}$ and $(1,-1)^{T}$ in the forward and backward directions respectively.

Fig. S3(a) and (b) show the eigenstate evolution of the system with $C=0$ during forward and backward propagation with the change of $\left|\frac{\rho}{\chi}\right|$, respectively. When$\left|\frac{\rho}{\chi}\right|=\infty(\rho\neq0,\chi=0)$, the eigenstates of forward and backward propagation are $(1,0)^{T}$and $(0,1)^{T}$, where the fast eigenstate is $(0,1)^{T}$ and the slow eigenstate is $(1,0)^{T}$[1]. When, with decreasing$\left|\frac{\rho}{\chi}\right|$, the two eigenstates $|\psi_{\pm}^{R,f_{3}}\rangle$ converge from $(1,0)^{T}$ and $(0,1)^{T}$ to $(1,1)^{T}$, while $|\psi_{\pm}^{R,b_{3}}\rangle$ converges from $(1,0)^{T}$ and $(0,1)^{T}$ to $(1,-1)^{T}$, respectively, represented by the yellow trajectory on the sphere. When $\left|\frac{\rho}{\chi}\right|=1$, the EP occurs in both the forward and backward propagation systems. That is, the eigenstates of forward and backward propagation degenerate at $(1,1)^{T}$ and$(1,-1)^{T}$, respectively. As $\left|\frac{\rho}{\chi}\right|$ continues to decrease, when$\left|\frac{\rho}{\chi}\right|<1$, the eigenstates $|\psi_{\pm}^{R,f_{3}}\rangle$ and $|\psi_{\pm}^{R,b_{3}}\rangle$ split from the $(1,1)^{T}$ and $(1,-1)^{T}$ to the $(1,i)^{T}$ and $(1,-i)^{T}$ states respectively, represented by the blue trajectory on the sphere. When $\left|\frac{\rho}{\chi}\right|=0(\rho=0,\chi\neq0)$, the eigenstates of forward propagation are $(1,i)^{T}$and $(1,-i)^{T}$, where the gain eigenstate is $(1,i)^{T}$ and the loss eigenstate is $(1,-i)^{T}$. For $\left|\frac{\rho}{\chi}\right|=0(\rho=0,\chi\neq0)$ in the backward propagation direction, the eigenstates are $(1,i)^{T}$and $(1,-i)^{T}$, where the gain eigenstate is $(1,-i)^{T}$ and the loss eigenstate is $(1,i)^{T}$. Hence, the eigenstate evolution process shows that the eigenstates of the proposed system are different for forward and backward propagation when chirality exists.


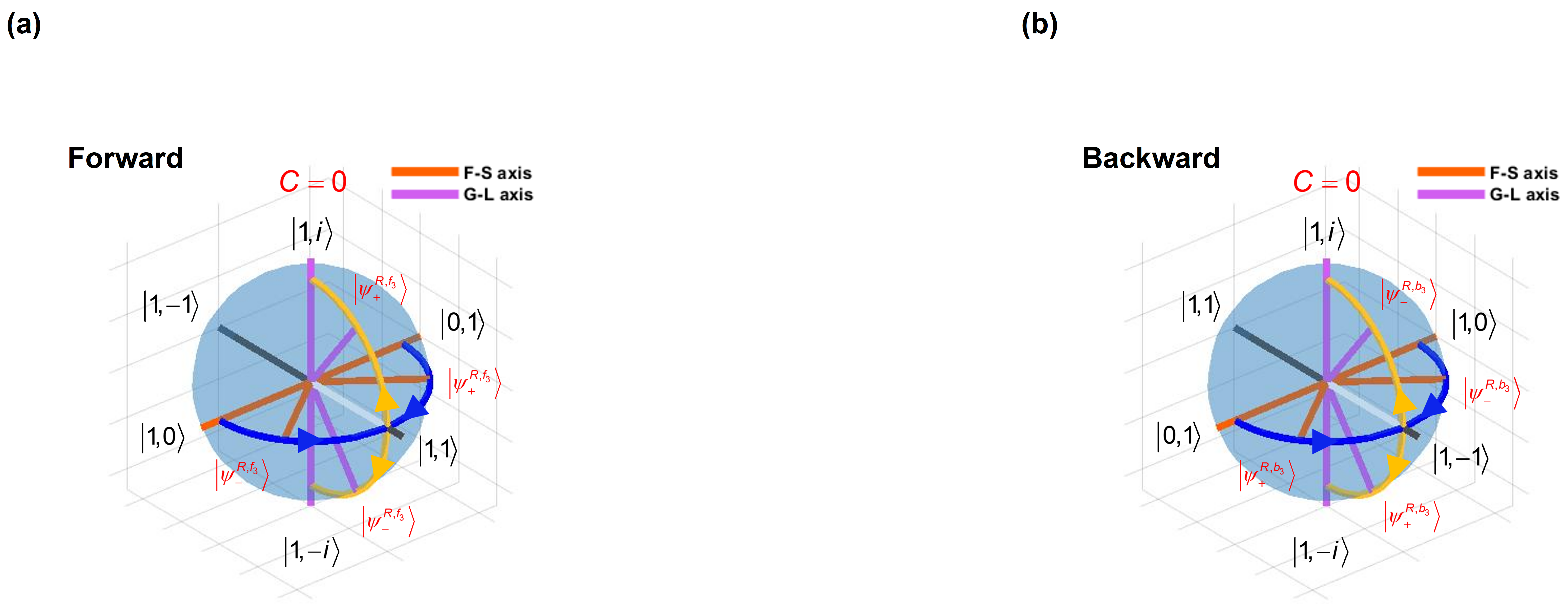


Fig. S3 Poincaré spheres showing the evolution of eigenstates of light passing through such a special 3D chirality non-Hermitian system with $C=0$in both the forward (a) and backward (b) directions.

Then, for several systems with a specific $\left|\frac{\rho}{\chi}\right|$, the evolution of light polarization states during forward and backward propagation is analyzed. When forward propagation is considered, in the broken phase of $\left|\frac{\rho}{\chi}\right|=\frac{2\sqrt{3}}{3}$, the eigenvalues are two different complex numbers in Eq. (S9) and the corresponding eigenstates are two nonorthogonal linear polarizations that experience equal gain and loss. The polarization dynamics in this case are depicted on the Poincaré sphere, as shown in Fig. S4(a). Since $|\psi_{+}^{R,f_{3}}\rangle$ is the gain eigenstate, and $|\psi_{-}^{R,f_{3}}\rangle$is the loss eigenstate, any polarization eigenstate except the loss eigenstate $|\psi_{-}^{R,f_{3}}\rangle$ will be amplified and rotated toward the gain eigenstate $|\psi_{+}^{R,f_{3}}\rangle$, as described in the yellow and purple trajectories on the Poincaré sphere. Nevertheless, in the exact phase of $\left|\frac{\rho}{\chi}\right|=\frac{1}{3}$, the eigenvalues are two different real numbers, and the corresponding eigenstates are two elliptic polarization states. The other polarization states precess in circles about nonorthogonal eigenstates $|\psi_{\pm}^{R,f_{3}}\rangle$**,** as shown in Fig. S4(b). For $\left|\frac{\rho}{\chi}\right|=\frac{2\sqrt{3}}{3}$in the backward propagation direction, the result is similar to that of forward propagation, with the exception of the loss eigenstate$|\psi_{-}^{R,b_{3}}\rangle$, all polarization states are amplified toward the gain eigenstate $|\psi_{+}^{R,b_{3}}\rangle$. However, a notable distinction arises from the fact that the forward and backward polarization eigenstates in our system are different, so the gain eigenstate$|\psi_{+}^{R,b_{3}}\rangle$ and loss eigenstate $|\psi_{-}^{R,b_{3}}\rangle$ are different from those of forward propagation, resulting in an opposite direction of the evolution trajectory of the eigenstates compared with forward propagation, as shown in Fig. S4(d). Moreover, for $\left|\frac{\rho}{\chi}\right|=\frac{1}{3}$ in backward propagation, the result is also analogous to that in forward propagation. The polarization state precesses in circles around a pair of nonorthogonal states$|\psi_{\pm}^{R,b_{3}}\rangle$. The yellow trajectory on the sphere in Fig. S4(e) is shown. Similarly, the direction of rotation of the polarization state is opposite to that of forward propagation. Eq. (S9) and Eq. (S12) indicate that when $\left|\frac{\rho}{\chi}\right|=1$, EP occurs in both forward propagation and backward propagation, where the eigenstates coalesce into $(1,1)^{T}$ and $(1,-1)^{T}$ in the forward and backward directions respectively. And no matter what polarization state is incident, the polarization of light will evolve toward the eigenstate, as shown in Fig. S4(c) and Fig. S4(f). At this time, the polarization of light propagating forward and backward will evolve toward two vertically degenerate eigenstates. Therefore, we can conclude that due to the distinct eigenstates of forward and backward propagation of 3D chiral non-Hermitian systems with $C=0$, polarized light will experience different polarization evolutions when transmitted forward and backward, resulting in asymmetric state switching. When the system is at the EP, the light propagating forward and backward will end up in two orthogonal polarization states after a sufficiently long evolutionary distance. On this basis, an omni-polarizer is realized.


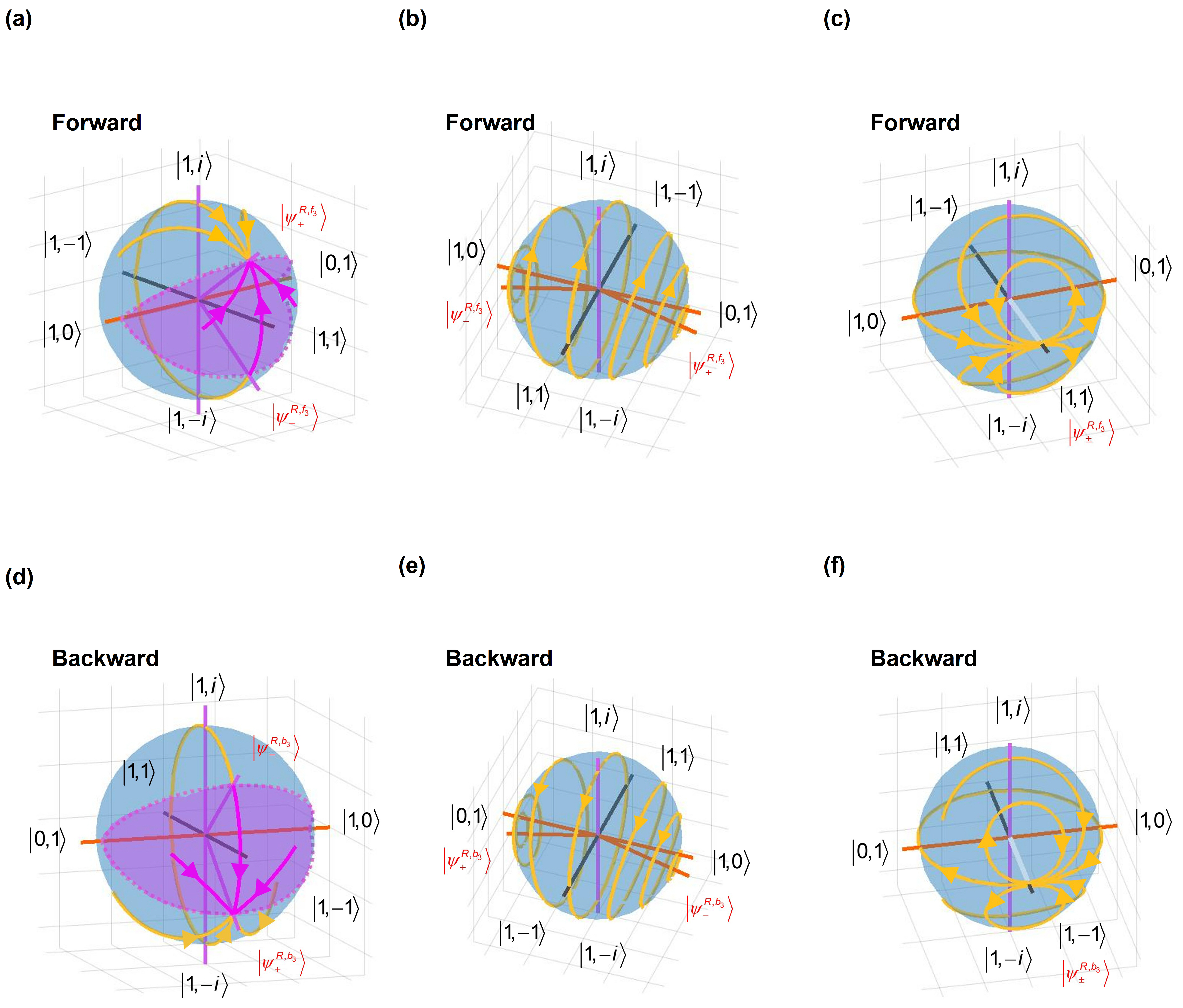


Fig. S4 Poincaré spheres describe the polarization dynamics of light passing through a special 3D chirality non-Hermitian system with $C=0$ in both the forward and backward directions for a specific $\left|\frac{\rho}{\chi}\right|$, where (a), (d) $\left|\frac{\rho}{\chi}\right|=\frac{2\sqrt{3}}{3}$, (b), (e) $\left|\frac{\rho}{\chi}\right|=\frac{1}{3}$and (c), (f) $\left|\frac{\rho}{\chi}\right|=1$.

**S2: Simulation of the 3D chiral non-Hermitian system at the EP**

Next, to further explore the characteristics of the 3D chiral non-Hermitian system at the EP, the commercial software COMSOL Multiphysics was employed to simulate the configuration of the theoretical model at the EP in free space. The simulation utilized the beam envelope method within the wave optical module to construct a cuboid space model. Then, we set each parameter in Eq. (1) as $\varepsilon_{0}=4,\gamma=\mu=\rho=\chi=0.01$. Scattering boundary conditions were applied to the boundaries perpendicular to the z-axis (propagation direction). Periodic boundary conditions were applied to the boundaries perpendicular to the x-axis and y-axis. Fig. S5(a) shows a schematic of an omni-polarizer on the basis of our theoretical derivation and simulation results. The omni-polarizer is fundamentally different from the conventional polarizer because it exhibits different propagation orthogonal polarization states when propagating forward and backward. After a sufficiently long propagation distance of 3000 µm, the output polarizations for an arbitrary input polarization state (300 input polarization states were simulated) evolve into the eigenstates $(1,\pm1)^{T}$ for forward and backward propagation, as shown in Fig. S5(a). All the electric field polarizations are almost the same, as illustrated in Fig. S5(b) (±45° linear polarization), which corresponds to the eigenstates$(1,\pm1)^{T}$. For a 3D chiral non-Hermitian system with any C value, asymmetric state switching and omni-polarizer action can be achieved. For different C values, only the evolution trajectories of the systems are different. Compared with the general 3D chiral non-Hermitian system, the special case would have a more special polarization state evolution trajectory, which would be more distinctive. We further explore the behavior of the system at an EP by simulating the system at $C=\infty$. Then, we set each parameter in Eq. (1) as $\varepsilon_{0}=4,\gamma=\mu=0.01$. In addition, to further illustrate this characteristic, we select horizontal linear polarization (HLP), vertical linear polarization (VLP), left circular polarization (LCP) and right circular polarization (RCP) as the input polarization states. Then, their dynamic polarization evolutions for forward and backward propagation are simulated at a wavelength of 1550 nm and depicted on a Poincaré sphere, as shown in Fig. 5(b) and Fig. 5(c). The solid data points in Fig. S5(c) and (d) represent the polarization state after a fixed evolutionary length of 13 µm. As the evolution of the polarization state approaches the eigenstate, its evolution rate becomes extremely slow. Thus, twice the previous evolutionary length is used to achieve rapid evolution near the EP, as shown in Fig. S5(c) and (d) for the hollow data points. As indicated by the arrows, all these input polarization states for forward and backward propagation rotate toward the eigenstates$(1,\pm1)^{T}$following the expected evolution path. Our simulation results further confirm our previous theoretical derivation. After a sufficiently long propagation distance, an arbitrary input polarization state evolves into two degenerate eigenstates that are perpendicular to each other for forward and backward propagation, which can act as an omni-polarizer.


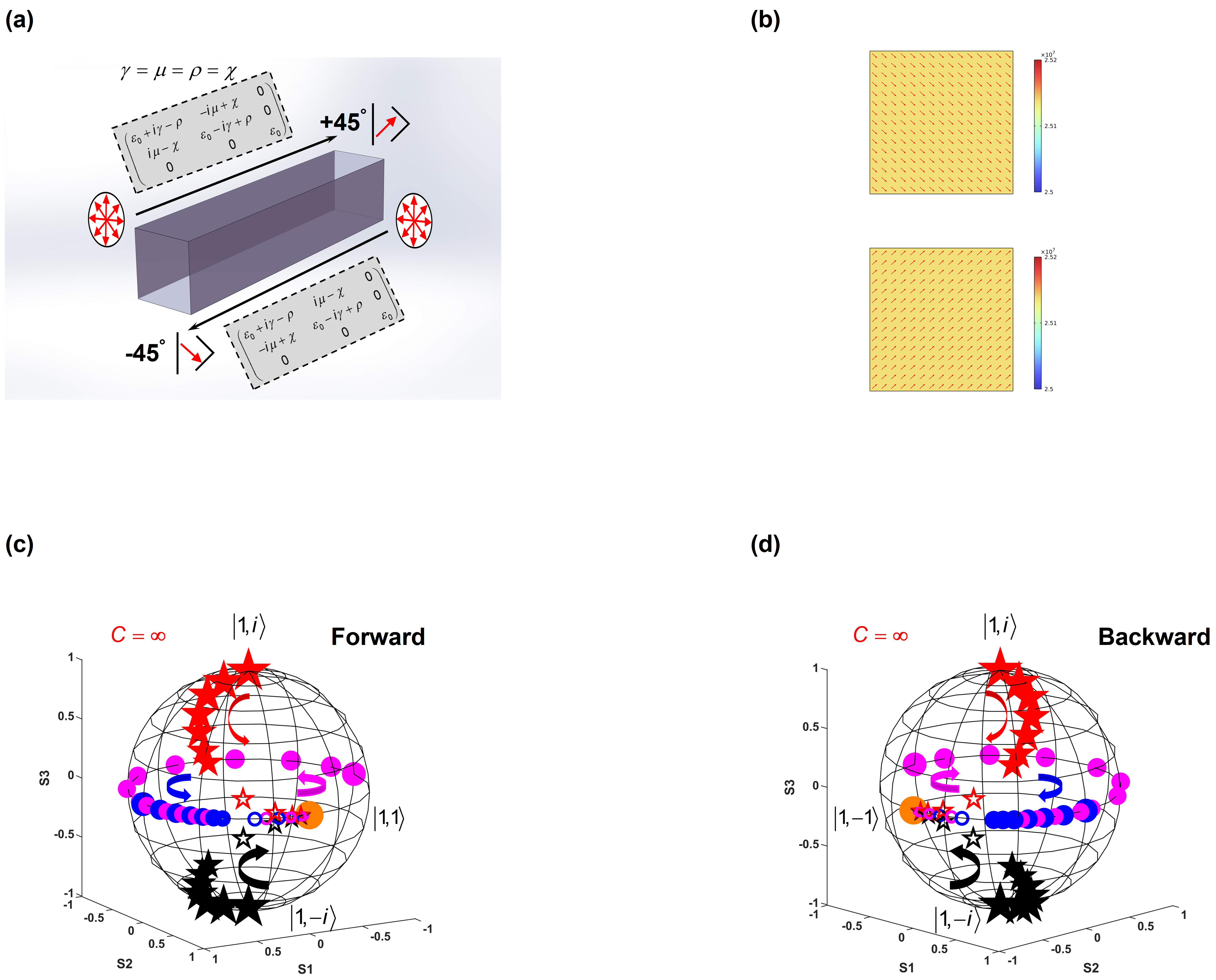


Fig. S5 (a) Schematic diagram of the omni-polarizer during forward and backward propagation. The gray area represents the permittivity-tensor of a general 3D chiral non-Hermitian system propagating forward and backward, where the direction-dependent polarization conversions are indicated by red arrows. (b) The corresponding two eigenpolarizations and their electric field polarization diagrams. (c) and (d) Poincaré sphere tracks of polarization evolution as several selected input states passing through the 3D chiral non-Hermitian system with $C=\infty$ are simulated at the EP for the forward and backward directions, and the direction of the evolution trajectory is indicated by arrows.

**S3: Derivation of the Jones matrix for the theoretical system**

We assume that the eigenvector of the permittivity tensor is not degenerate, and arbitrarily choose a set of linearly independent basis vectors, set $e_{1}$ and $e_{2}$, and the eigenstates of the system are expressed as $\nu_{eig1}e^{i\beta_{1}t}=A_{11}e^{i\beta_{1}t}e_{1}+A_{12}e^{i\beta_{1}t}e_{2}$and $\nu_{eig2}e^{i\beta_{2}t}=A_{21}e^{i\beta_{2}t}e_{1}+A_{22}e^{i\beta_{2}t}e_{2}$. Generally, we choose the base vector as the x polarized light and the y polarized light, and the corresponding base vectors are expressed as$\left(1,0\right)^{T}$ and $\left(0,1\right)^{T}$, respectively. From the relation of $\nu_{eig1}=A_{11}e_{1}+A_{12}e_{2}\equiv\left[A_{11},A_{12}\right]^{T}_{LP}$and $\nu_{eig2}=A_{21}e_{1}+A_{22}e_{2}\equiv\left[A_{21},A_{22}\right]^{T}_{LP}$, a forward Jones matrix between the incident wave$E_{inc}=\left[E_{xI},E_{yI}\right]^{T}$and the transmitted wave$E_{tra}=\left[E_{xT},E_{yT}\right]^{T}$ in the LP basis is obtained as [1]

$$\left[\begin{matrix} E_{xT} \\ E_{yT} \end{matrix}\right]=\frac{1}{A_{11}A_{22}-A_{12}A_{21}}\left[\begin{matrix} A_{11}e^{-i\beta_{1}d} & A_{21}e^{-i\beta_{2}d} \\ A_{12}e^{-i\beta_{1}d} & A_{22}e^{-i\beta_{2}d} \end{matrix}\right]\left[\begin{matrix} A_{22} & -A_{21} \\ -A_{12} & A_{11} \end{matrix}\right]\left[\begin{matrix} E_{xI} \\ E_{yI} \end{matrix}\right]$$

In addition, plugging in the eigenstate of the system, Eq. (S14) can be re-expressed as$E_{tra}=t^{f}E_{inc}$, where

Where , ,$\beta_{1,2}=\frac{2\pi\sqrt{\varepsilon^{f_{1}}_{1,2}}}{\Lambda_{0}}$,$\lambda'_{pt}=\sqrt{(\rho-i\gamma)^{2}-(\chi-i\mu)^{2}}$, and$\Lambda_{0}$ represents our chosen laser wavelength. In addition, when the system is at the EP, $\rho=\chi,\gamma=\mu$, by the limit

We can rewrite the above Jones matrix $t^{f_{1}}$ as

We can subsequently use the same method to obtain the backward Jones matrix $t^{b_{1}}$, as shown below

Similarly, when the system is at the EP, the backward Jones matrix $t^{b_{1}}$ can be expressed as

To more intuitively recognize the EP and non-EP cases, a rotation matrix *S* is employed to transform the coordinate axis $45^{0}$ clockwise around the z-axis, that is

$$t'=S^{-1}tS$$

Where

$$S=\frac{1}{\sqrt{2}}\left(\begin{matrix} 1 & -1 \\ 1 & 1 \end{matrix}\right)$$

In addition, to distinguish the coordinates before and after the rotation, we use $t=\left\{t_{ij}\right\}$ for the pre-rotation and $t'=\left\{t'_{i'j'}\right\}$ for the post-rotation. Thus, the Jones matrices $t^{f_{1}}$ and $t^{b_{1}}$ are transformed into

and

Moreover, the Jones matrices of the system at an EP can be rewritten as

And

**S4. The Experimental setup and** **structure parameter of the large area extrinsic chiral metasurface**

The extrinsic chiral metasurface utilized in this study comprises a rectangular lattice array of gold split-ring resonators (SRRs) supported by a glass substrate, as depicted in Fig. S6. The gold split-ring resonators were fabricated using electron beam lithography (EBL) (Tianjin H-Chip Technology Group Corporation). The lattice period of the metasurface is Px = 1000 nm in the x-direction and Py = 650 nm in the y-direction. The metasurface extends over the (x, y) plane with the symmetry axis of the SRRs oriented along the y direction. The structure is illuminated at an oblique angle by circularly polarized light traveling in the z-direction. The illustration to the right of Fig. S6 displays a scanning electron microscope (SEM) image showcasing the fabrication of the metasurface. The gold split-ring resonator possesses a U-shaped structure with a radius of R = 350 nm, a width of w = 200 nm, and a thickness of approximately 100 nm [3].


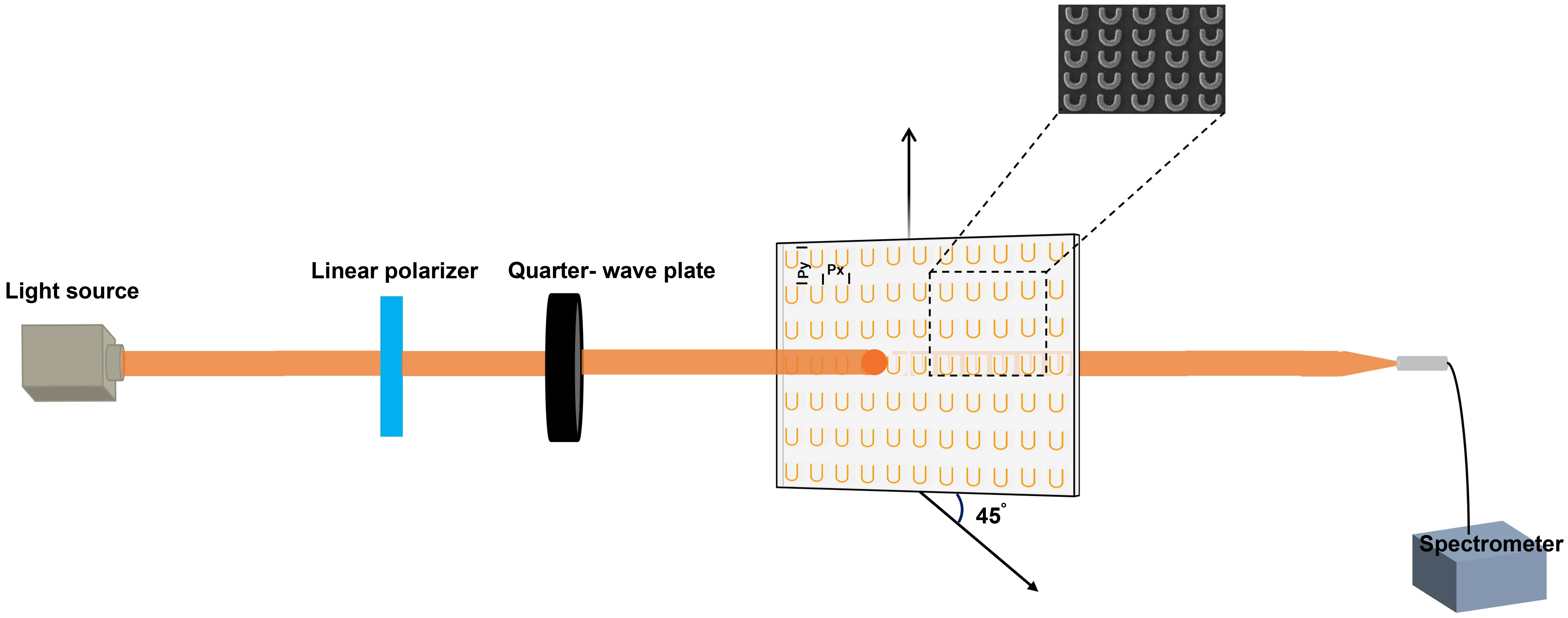


Fig. S6 The measurement setup for the transmitted circular dichroism (CD) and of the extrinsic chiral metasurface.

**S5. Preparation of the Dichroic** **Material**

The preparation process of the dichroic material is as follows:

1. First 10 g of polyvinyl alcohol was weighed and placed in a three-mouth flask, 400 ml of distilled water was added, and the mixture was boiled in a water bath, and stirred constantly with a stirrer, until it was completely dissolved in a gel.
2. Then, 0.5 g of iodine was added to a beaker, 50 ml of distilled water was added, and 1.0 g of potassium iodide was added. The mixture was stirred until completely dissolved.
3. When the dissolution concentration in (1) is reduced to 60~70 °C, the iodine dissolution concentration that has been matched is slowly increased, and the solution is stirred while being added and stirred evenly until it is completely added.
4. The above mixture was poured onto a clean, flat glass plate and flattened with a glass rod. The above ingredients can produce a material of approximately 1500 cm^2^.
5. The glass plate obtained from (4) was placed in a clean, dry and dust-free room, and then allowed to dry naturally to form a film.
6. The dry film was removed from one corner with a dull knife and gently pulled with the hand, so that the whole film from the glass plate was removed, according to the need to cut it to the appropriate size.
7. The film was stretched. The tensile temperature was maintained at 60~70 °C. It can be stretched on the material testing machine, and an electric furnace is placed on the testing machine workbench. The electric furnace can be powered off after heating, and the waste heat can be stretched. When the amount of stretching is 3 to 4 times, it is appropriate. It can also be drawn by a specially designed drawing machine.
8. The stretched film was soaked in anhydrous ethanol for approximately 15 min to wash away the residual iodine, potassium iodide and fingerprints on the surface.
9. The prepared film material is etched with a reactive ion beam to achieve precise thinning to obtain the desired thickness of the material.
10. The above prepared film material is thin and easy to deform, and it is necessary to clamp it with two well-transparent glass sheets and place it in the sample box.

Thus, dichroic materials with controllable thickness and angle (the angle between the optical axis of a dichroic material and the x- polarization direction) are prepared. By adjusting the thickness and angle of a dichroic material and the parameters of other optical components, the experimental system can be adjusted to a state very close to the EP. Finally, in the experiment, we obtained a 3D chiral non-Hermitian system near an EP when the thickness of the dichroic material was approximately 0.176 mm and the angle deviated from the horizontal plane by approximately 42.5°.

**S6. The experimental method for measuring the Jones matrix of the entire non-Hermitian system.**

Here, we briefly introduce the relationships between Jones matrix, Stokes vector, and coherence matrix. Then, a completely polarized light beam can be represented by a Jones vector, typically written as follows:

$$\vec{E}=\left(\begin{matrix} E_{x} \\ E_{y} \end{matrix}\right)=\left(\begin{matrix} A_{x} \\ A_{y}\exp(i\delta) \end{matrix}\right)$$

where, A_x_ and A_y_ are the electric field intensity in the x and y directions respectively. $\delta$ is the phase difference in the x and y directions. The Stokes vector is usually expressed as:

$$S\text{=}\left[\begin{matrix} S_{0} \\ S_{1} \\ S_{2} \\ S_{3} \end{matrix}\right]=\left[\begin{matrix} \left|E_{x}\right|^{2}+\left|E_{y}\right|^{2} \\ \left|E_{x}\right|^{2}-\left|E_{y}\right|^{2} \\ 2A_{x}A_{y}\cos\delta\\ 2A_{x}A_{y}\sin\delta\end{matrix}\right]$$

where, S_0_ corresponds to the total light intensity of the polarized light beam, S_1_ describes the difference in light intensity between horizontally polarized and vertically polarized light, S_2_ involves the comparison of light intensity between 45^0^ linearly polarized and 135^0^ linearly polarized light, while S_3_ distinguishes the difference in light intensity between right-handed circularly polarized and left-handed circularly polarized light. The coherence matrix can be represented by the elements A_x_, A_y_, $\exp(i\delta)$ in the Jones matrix

$$M=\left(\begin{matrix} A_{x}^{2} & A_{x}A_{y}\exp(-i\delta) \\ A_{x}A_{y}\exp(i\delta) & A_{y}^{2} \end{matrix}\right)$$

By solving Eq. (S26) and Eq. (S27) simultaneously, Eq. (S28) can be rewritten as

$$M=\frac{1}{2}\left(\begin{matrix} S_{0}+S_{1} & S_{2}-iS_{3} \\ S_{2}+iS_{3} & S_{0}-S_{1} \end{matrix}\right)$$

Next, we assume that under the basis vectors of x-linearly polarized light and y-linearly polarized light, the Jones matrix of a certain optical element can be represented as follows:

$$G=\left(\begin{matrix} A & B \\ C & D \end{matrix}\right)$$

The coherent matrix of the polarized light after passing through the optical element can be represented as follows:

$$M_{out}=GM_{in}G^{\dagger}$$

Then, we set the total light intensity in the Stokes parameter of the incident light to be $S_{0}=1$, the coherence matrix $M(W_{in})$ of the incident light is expressed as:

$$M(W_{in})=\frac{1}{2}\left(\begin{matrix} 1+S_{1}(W_{in}) & S_{2}(W_{in})-iS_{3}(W_{in}) \\ S_{2}(W_{in})+iS_{3}(W_{in}) & 1-S_{1}(W_{in}) \end{matrix}\right)$$

For the Stokes parameters of the output light, since the intensity of the light may change after incidence, the coherence matrix is expressed as follows:

$$M(W_{out})=\frac{1}{2}\left(\begin{matrix} S_{0}(W_{out})+S_{1}(W_{out}) & S_{2}(W_{in})-iS_{3}(W_{out}) \\ S_{2}(W_{out})+iS_{3}(W_{out}) & S_{0}(W_{out})-S_{1}(W_{out}) \end{matrix}\right)$$

Next, we assume that the incident light is x-polarized light and the corresponding output light being $W_{1,out}$. According to Eq. (S33), the coherence matrix of the x-polarized light after passing through the optical element can be expressed as:

$$\begin{matrix} M_{1,out}=\left(\begin{matrix} A & B \\ C & D \end{matrix}\right)\left(\begin{matrix} 1 & 0 \\ 0 & 0 \end{matrix}\right)\left(\begin{matrix} A & B \\ C & D \end{matrix}\right)^{\dagger} \\ =\left(\begin{matrix} A & B \\ C & D \end{matrix}\right)\left(\begin{matrix} 1 & 0 \\ 0 & 0 \end{matrix}\right)\left(\begin{matrix} A^{*} & C^{*} \\ B^{*} & D^{*} \end{matrix}\right) \\ =\left(\begin{matrix} \left|A\right|^{2} & AC^{*} \\ A^{*}C & \left|C\right|^{2} \end{matrix}\right) \end{matrix}$$

Therefore, the following relationship can be obtained

$$\begin{matrix} \left|A\right|=\sqrt{1/2*(S_{0}(W_{1,out})+S_{1}(W_{1,out}))} \\ \left|C\right|=\sqrt{1/2*(S_{0}(W_{1,out})-S_{1}(W_{1,out}))} \\ \delta_{C}-\delta_{A}=Arg(S_{3}(W_{1,out})/S_{2}(W_{1,out})) \end{matrix}$$

For the incident light is y-polarized light and the corresponding output light being $W_{2,out}$. Analogously, the coherence matrix of the y-polarized light after passing through the optical element can be expressed as:

$$\begin{matrix} M_{2,out}=\left(\begin{matrix} A & B \\ C & D \end{matrix}\right)\left(\begin{matrix} 0 & 0 \\ 0 & 1 \end{matrix}\right)\left(\begin{matrix} A & B \\ C & D \end{matrix}\right)^{\dagger} \\ =\left(\begin{matrix} A & B \\ C & D \end{matrix}\right)\left(\begin{matrix} 0 & 0 \\ 0 & 1 \end{matrix}\right)\left(\begin{matrix} A^{*} & C^{*} \\ B^{*} & D^{*} \end{matrix}\right) \\ =\left(\begin{matrix} \left|B\right|^{2} & BD^{*} \\ B^{*}D & \left|D\right|^{2} \end{matrix}\right) \end{matrix}$$

Therefore, the following relationship can be obtained

$$\begin{matrix} \left|B\right|=\sqrt{1/2*(S_{0}(W_{2,out})+S_{1}(W_{2,out}))} \\ \left|D\right|=\sqrt{1/2*(S_{0}(W_{2,out})-S_{1}(W_{2,out}))} \\ \delta_{D}-\delta_{B}=Arg(S_{3}(W_{2,out})/S_{2}(W_{2,out})) \end{matrix}$$

For the incident light is 45^0^ polarized light and the corresponding output light being $W_{3,out}$. Analogously, the coherence matrix of the 45^0^ polarized light after passing through the optical element can be expressed as:

$$\begin{matrix} M_{3,out}=\frac{1}{2}\left(\begin{matrix} A & B \\ C & D \end{matrix}\right)\left(\begin{matrix} 1 & 1 \\ 1 & 1 \end{matrix}\right)\left(\begin{matrix} A & B \\ C & D \end{matrix}\right)^{\dagger} \\ =\frac{1}{2}\left(\begin{matrix} A & B \\ C & D \end{matrix}\right)\left(\begin{matrix} 1 & 1 \\ 1 & 1 \end{matrix}\right)\left(\begin{matrix} A^{*} & C^{*} \\ B^{*} & D^{*} \end{matrix}\right) \\ =\frac{1}{2}\left(\begin{matrix} \left|A\right|^{2}+\left|B\right|^{2}+2\left|A\right|\left|B\right|\cos(\delta_{A}-\delta_{B}) & AC^{*}+AD^{*}+BC^{*}+BD^{*} \\ A^{*}C+A^{*}D+B^{*}C+B^{*}D & \left|C\right|^{2}+\left|D\right|^{2}+2\left|C\right|\left|D\right|\cos(\delta_{C}-\delta_{D}) \end{matrix}\right) \end{matrix}$$

Therefore, the following relationship can be obtained

$$AD^{*}+BC^{*}=S_{2}(W_{3,out})-iS_{3}(W_{3,out})-AC^{*}-BD^{*}$$

For the incident light is right-handed polarized light and the corresponding output light being $W_{4,out}$. Analogously, the coherence matrix of the right-handed polarized light after passing through the optical element can be expressed as:

$$\begin{matrix} M_{3,out}=\frac{1}{2}\left(\begin{matrix} A & B \\ C & D \end{matrix}\right)\left(\begin{matrix} 1 & -i \\ i & 1 \end{matrix}\right)\left(\begin{matrix} A & B \\ C & D \end{matrix}\right)^{\dagger} \\ =\frac{1}{2}\left(\begin{matrix} A & B \\ C & D \end{matrix}\right)\left(\begin{matrix} 1 & -i \\ i & 1 \end{matrix}\right)\left(\begin{matrix} A^{*} & C^{*} \\ B^{*} & D^{*} \end{matrix}\right) \\ =\frac{1}{2}\left(\begin{matrix} \left|A\right|^{2}+\left|B\right|^{2}+2\left|A\right|\left|B\right|\sin(\delta_{A}-\delta_{B}) & AC^{*}+BD^{*}+iBC^{*}-iAD^{*} \\ A^{*}C+B^{*}D+iA^{*}D-iB^{*}C & \left|C\right|^{2}+\left|D\right|^{2}+2\left|C\right|\left|D\right|\sin(\delta_{C}-\delta_{D}) \end{matrix}\right) \end{matrix}$$

Therefore, the following relationship can be obtained

$$iBC^{*}-iAD^{*}=S_{2}(W_{4,out})-iS_{3}(W_{4,out})-AC^{*}-BD^{*}$$

From the above, we can obtain the following derivation:

$$\begin{matrix} AD^{*}=\frac{1}{2}\left[S_{2}(W_{3,out})+S_{3}(W_{4,out})-i(S_{3}(W_{3,out})-S_{2}(W_{4,out}))-(1+i)AC^{*}-(1+i)BD^{*}\right] \\ BC^{*}=\frac{1}{2}\left[S_{2}(W_{3,out})-S_{3}(W_{4,out})-i(S_{3}(W_{3,out})+S_{2}(W_{4,out}))-(1-i)AC^{*}-(1-i)BD^{*}\right] \end{matrix}$$

Then, we can derive the following relation

$$\begin{matrix} \delta_{A}-\delta_{D}=Arg(AD^{*}) \\ \delta_{B}-\delta_{C}=Arg(BC^{*}) \end{matrix}$$

Finally, we can derive the Jones matrix：

$$G'=\left(\begin{matrix} \left|A\right| & \left|B\right|\exp(i(\delta_{B}-\delta_{A})) \\ \left|C\right|\exp(i(\delta_{C}-\delta_{A})) & \left|D\right|\exp(i(\delta_{D}-\delta_{A})) \end{matrix}\right)$$

**S7. The specific steps of the simulated experiment adjustment process through numerical calculation**

The specific steps of the computational simulation are as follows:

1. Set initial values for four parameters ($\alpha_{Di}$,$\theta_{Di}$,$\theta_{Bib}$,$\beta_{Bib}$).
2. First iteration. (1) Let the parameter $\alpha_{Di}$ take 1200 points uniformly on both sides of its initial value, where the interval of each point is 0.00005. Then, fix the remaining three parameters and the $\alpha_{Di}$corresponding to the point closest to the origin (EP) is selected to replace the initial value for the next operation. (2) Let the parameter $\theta_{Di}$ take 1200 points uniformly on both sides of its initial value, where the interval of each point is $\pi/11520$. Then, fix the remaining three parameters and the $\theta_{Di}$ corresponding to the point closest to the origin (EP) is selected to replace the initial value for the next operation. (3) Let the parameter $\theta_{Bib}$ take 1200 points uniformly on both sides of its initial value, where the interval of each point is $\pi/11520$. Then, fix the remaining three parameters and the $\theta_{Bib}$ corresponding to the point closest to the origin (EP) is selected to replace the initial value for the next operation. (4) Let the parameter $\beta_{Bib}$ take 1200 points uniformly on both sides of its initial value, where the interval of each point is $\pi/11520$. Then, fix the remaining three parameters and the $\beta_{Bib}$ corresponding to the point closest to the origin (EP) is selected to replace the initial value for the next operation. After such a cycles of taking points (1), (2), (3) and (4), we can obtain a new set of four parameter values ($\alpha'_{Di}$,$\theta'_{Di}$,$\theta'_{Bib}$,$\beta'_{Bib}$) and a 3D coordinate point.
3. Iterative loop. Replace the values in step 1 with the values of the new set of four parameters ($\alpha'_{Di}$,$\theta'_{Di}$,$\theta'_{Bib}$,$\beta'_{Bib}$) obtained in step 2 as initial values, and then repeat the steps in step 2 for multiple iterations. The more iterations performed, the closer the final three-dimensional coordinate points get to (0, 0, 0).
4. Termination condition. Repeat iterations until convergence.

**S8. The simulation process involves tuning 3D chiral non-Hermitian systems with different initial Jones matrices to near an EP and studying the corresponding evolution of polarization states when propagating forward.**

To further demonstrate the broad applicability of the proposed method, we achieve 3D non-Hermitian systems with different initial Jones matrices (corresponding to different initial 3D coordinate points and $g'$ ) to near an EP through a large number of computational simulations. The results of computational simulations indicate that all simulated 3D chiral non-Hermitian systems with different initial Jones matrices can achieve near an EP of $(1,1)^{T}$ when propagating forward (The backward propagation is all towards $(1,-1)^{T}$, and take forward propagation as an example here). However, their 3D coordinate points and evolution trajectories of polarization states are different. Fig.S7 (a) and (b) show the evolution processes of 3D coordinate points and the evolution trajectories of several input polarization states for a 3D chiral non-Hermitian systems with $\text{g}'\text{ = 0.1564+1.236i}$ when propagating forward. Fig.S7 (c) and (d) show the evolution processes of 3D coordinate points and the evolution trajectories of several input polarization states for a 3D chiral non-Hermitian systems with $\text{g}'\text{ = -0.717-0.2696i}$ when propagating forward. Fig.S7 (e) and (f) show the evolution processes of 3D coordinate points and the evolution trajectories of several input polarization states for three groups of 3D chiral non-Hermitian systems with$\text{g}'\text{ = -0.7133+0.5301i}$ when propagating forward. Therefore, through these three examples, it can be confirmed that we can tune 3D chiral non-Hermitian systems with different initial Jones matrices to achieve an EP when propagating forward, thus confirming that our proposed method has a wide applicability. Different values of $g'$ correspond to different values of *C*, resulting in distinct evolution paths of the polarization states, which is consistent with our theoretical predictions.

**
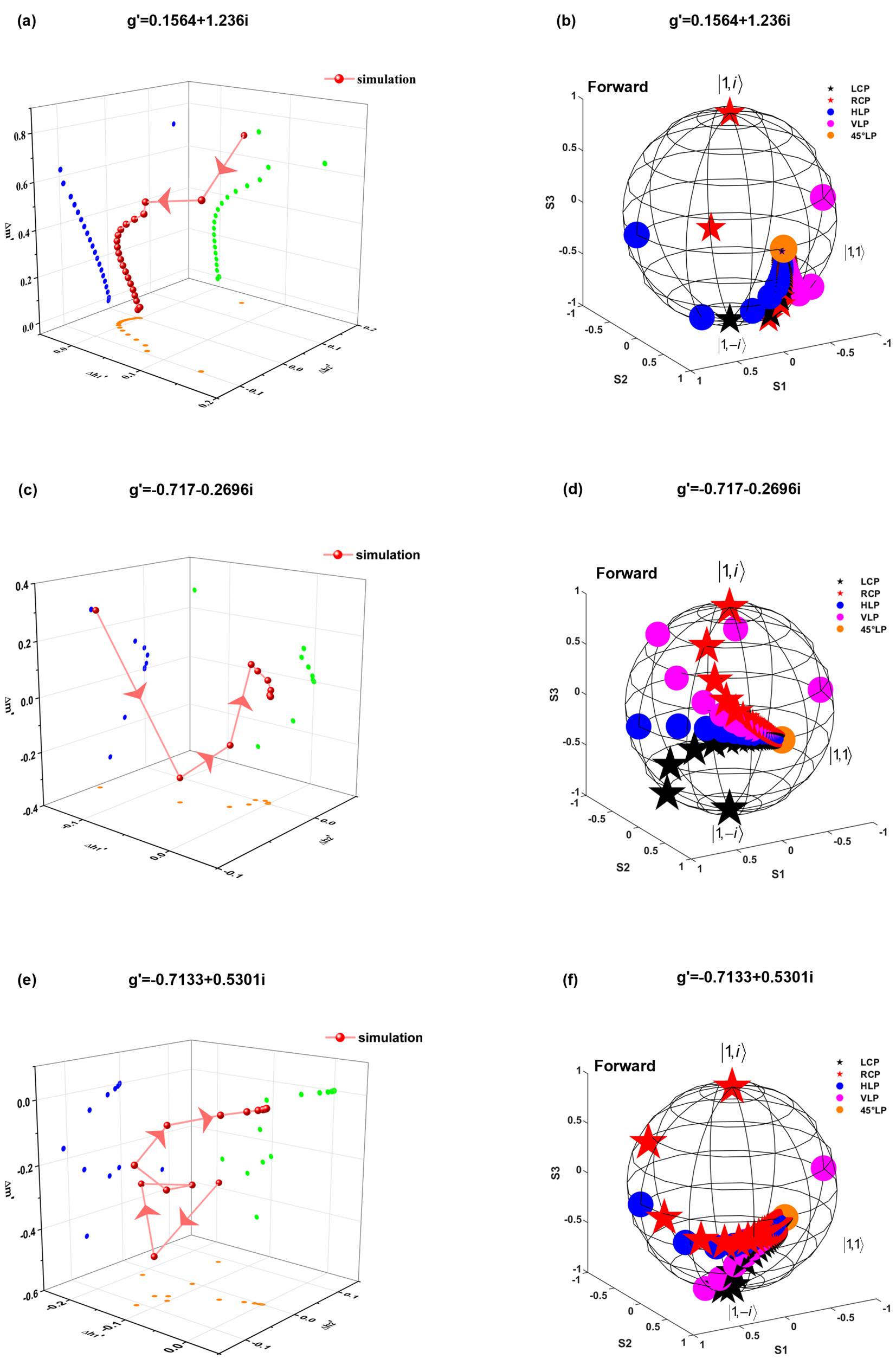
**

Fig. S7 The evolution processes of 3D coordinate points and the evolution trajectories of several input polarization states for three groups of 3D chiral non-Hermitian systems with different $g'$ values. The evolution processes of 3D coordinate points (a) and the evolution trajectories of several input polarization states (b) for 3D chiral non-Hermitian systems with$\text{g}'\text{ = 0.1564+1.236i}$. The evolution processes of 3D coordinate points (c) and the evolution trajectories of several input polarization states (d) for 3D chiral non-Hermitian systems with$\text{g}'\text{ = -0.717-0.2696i}$. The evolution processes of 3D coordinate points (e) and the evolution trajectories of several input polarization states (f) for 3D chiral non-Hermitian systems with $\text{g}'\text{ = -0.7133+0.5301i}$.

References

1 Yu, S. et al. Low-dimensional optical chirality in complex potentials. *Optica* **3**, 1025-1032, doi: 10.1364/optica.3.001025 (2016).

2 Buddhiraju, S. et al. Nonreciprocal metamaterial obeying time-reversal symmetry. *Physical Review Letters* **124**, 257403, doi: 10.1103/PhysRevLett.124.257403 (2020).

3 Fu, X. H. et al. Asymmetric reflection based on asymmetric coupling in single-layer extrinsic chiral metasurfaces. *Optics Express* **30**, 47124-47133, doi: 10.1364/OE.478073 (2022).
